# Large increase in global storm runoff extremes driven by climate and anthropogenic changes

**DOI:** 10.1038/s41467-018-06765-2

**Published:** 2018-10-22

**Authors:** Jiabo Yin, Pierre Gentine, Sha Zhou, Sylvia C. Sullivan, Ren Wang, Yao Zhang, Shenglian Guo

**Affiliations:** 1 State Key Laboratory of Water Resources and Hydropower Engineering Science, Wuhan University, Wuhan, 430072 China; 2 Department of Earth and Environmental Engineering, Columbia University, New York, NY 10027 USA; 3 Earth Institute, Columbia University, New York, NY 10025 USA; 4 School of Geography and Planning, Sun Yat-Sen University, Guangzhou, 510275 China

**Keywords:** Climate change, Hydrology, Hydrology, Natural hazards

## Abstract

Weather extremes have widespread harmful impacts on ecosystems and human communities with more deaths and economic losses from flash floods than any other severe weather-related hazards. Flash floods attributed to storm runoff extremes are projected to become more frequent and damaging globally due to a warming climate and anthropogenic changes, but previous studies have not examined the response of these storm runoff extremes to naturally and anthropogenically driven changes in surface temperature and atmospheric moisture content. Here we show that storm runoff extremes increase in most regions at rates higher than suggested by Clausius-Clapeyron scaling, which are systematically close to or exceed those of precipitation extremes over most regions of the globe, accompanied by large spatial and decadal variability. These results suggest that current projected response of storm runoff extremes to climate and anthropogenic changes may be underestimated, posing large threats for ecosystem and community resilience under future warming conditions.

## Abstract (web summary)

Storm runoff extremes dominate flash flood formation and generation, posing a grand threat to ecosystems and communities across the world. Here the authors show that current projected response of these storm runoff extremes to climate and anthropogenic changes are underestimated.

## Introduction

Because the saturation vapour pressure of water in the air is highly sensitive to temperature^1–3^, intensification of precipitation extremes by natural and anthropogenic changes (e.g., greenhouse gas emissions, irrigation, deforestation and grazing and land desertification) is expected and has been studied with both observational and modelling frameworks^4–7^. The Clausius–Clapeyron (C–C) scaling, characterising the increase of atmospheric moisture holding capacity with temperature (roughly 7% °C^−1^), has been widely used to evaluate extreme precipitation intensification with global warming^8–10^. Observations and simulations with climate models have reported a variety of scaling rates including strong super C–C scaling in mid-latitude regions but weak sub-C–C or negative rates in the tropics^11–14^.

The potential for extreme rainfall to intensify with climate change is of significant societal concern, and the flash floods attributed to these extreme-rain events are some of the most costly and dangerous natural hazards worldwide^15–17^. Flood hazards have caused substantial death tolls and property and agriculture losses across the world, rising over the past half century and exceeding $30 billion per year in the past decade^18^. Globally, almost 1 billion people are living in floodplains^19^, increasing the exposure to river flooding caused by extreme weather events and underscoring the urgency in comprehending and projecting these events. However, the expected responses of extreme storm runoff (i.e., fast flow, runoff removing the base flow contribution), dominating flash floods formation and generation, to warming temperature and precipitation extreme intensification had remained up to date unknown.

Here, a global scale hydrological analysis is performed, for the first time, to characterise the responses of storm runoff extremes to naturally and anthropogenically driven changes in temperature and atmospheric moisture content. Moreover, we assess the influence of decadal variability on the scaling of runoff extremes and temperature, and systematically compare this with changes in precipitation extremes. Observational daily runoff data are from the Global Runoff Data Centre (GRDC) datasets, and daily precipitation and near-surface air temperature data are from Global Summary of the Day (GSOD) dataset (Methods, Supplementary Fig. 1). We find that storm runoff extremes exhibit a super C–C scaling over most measured regions of the globe while precipitation extremes generally show a sub-C–C scaling, both of which are accompanied by spatial and decadal variability. These strong responses imply that more attention should be paid to the potentially underestimated response of storm runoff to climate and anthropogenic changes in order to improve our understanding and projection of flash flooding events and to improve community resilience.

## Results

### Global increase in extreme events

**Fig. 1:**
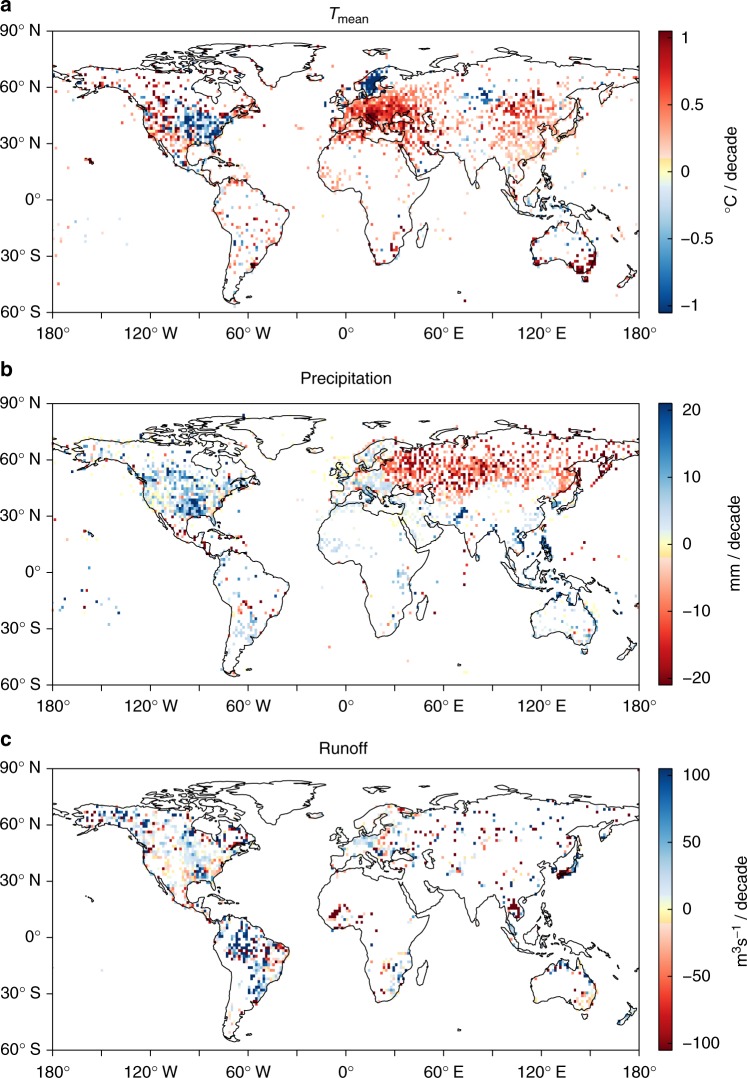
Global trend results for annual 99th percentile daily extremes during 1929–2017. **a**–**c** Trend of *T*_mean_ (**a**), precipitation (**b**) and runoff (**c**), respectively. White indicates grids with insufficient data or that the trend is insignificant at a 0.05 level

We first estimate the long-term trend during 1929–2017 for annual extremes, i.e., 99th and 95th percentile daily total precipitation, daily average runoff and corresponding daily mean temperature (*T*_mean_), daily maximum temperature (*T*_max_) and daily minimum temperature (*T*_min_). As expected, we find an overall positive trend of temperature at the global scale^20–22^, except in the midwestern US and north-western Europe, which exhibit distinct cooling trends (Fig. 1a and Supplementary Fig. 2). A recent regional study^23^ revealed that this cooling phenomenon might be attributed to intense agriculture and land management practices (Supplementary Fig. 3). Observational and modelling studies have demonstrated the ability of intense agriculture and irrigation to cool surface temperatures through increased evapotranspiration^24,25^. This cooling trend is attenuated when we use only temperatures on wet (i.e., rainy) days (Supplementary Fig. 4), because agriculture has larger cooling impacts on dry days when evapotranspiration is large.

Most rainfall stations outside of Russia show positive trends for precipitation extremes (Fig. 1b). Large increases are present in the midwestern US due to the increased moisture supply from irrigation^23^ and changes in mesoscale convective system (MCS) activity^26^. Agricultural intensification increases soil moisture and evapotranspiration^23–25^, leading to greater atmospheric moisture availability and is in line with the increasing trends of relative humidity (RH), specific humidity and moisture flux convergence in the midwestern US (Supplementary Fig. 5). A significant increase of the convective available potential energy in this region also makes the environment more favourable for convection and allows MCSs to grow larger^26,27^, thus resulting in a significant increase in the MCS rainfall volume^28^. A decreasing trend in precipitation dominates Russia, particularly a strong decreasing signal during 1961–1980, because moisture advection from the ocean is limited^29,30^, and decreased as a result of lower wind speeds and reduced specific humidity over Eurasia (Supplementary Fig. 6). The strong increase of precipitation, which prevails over Southeast Asia is consistent with previous findings, highlighting the change in regional atmospheric convergence there^31^. Most of global runoff stations show positive trends, although these are accompanied by large spatial variability in magnitude. The American continent indicates an overall increasing trend, while runoff extremes over the Sahel areas in Africa have declined (Fig. 1c); the fast flow extremes show similar changes (Supplementary Fig. 7). These changes in global extremes are more severe when we focus only on the more recent years, 1980–2017 (Supplementary Figs. 7–9). A more significant cooling trend is observed over the midwestern US and north-western European regions, and precipitation and runoff extremes show more distinct intensification over America, implying a stronger climate change impact in recent decades.

### Hook structure of extremes-temperature scaling

**Fig. 2:**
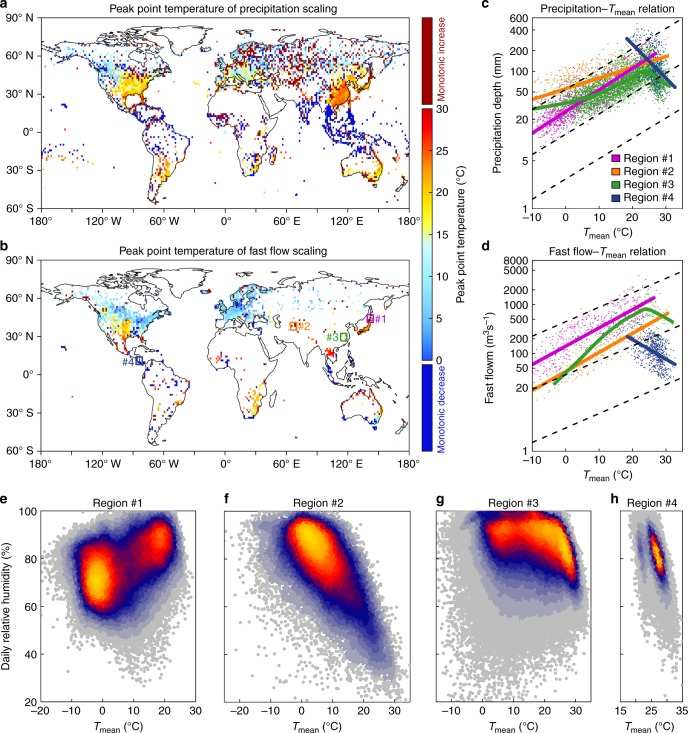
Peak point temperature for percentile 99th extreme with daily mean temperature and extremes and relative humidity varying with temperature over four example regions. **a**, **b** Peak point temperature of precipitation extremes (**a**), and fast flow extremes (**b**). **c**, **d** Relationship between *T*_mean_ with precipitation extremes (**c**), and fast flow extremes (**d**). **e**–**h** Relative humidity on wet day varying with *T*_mean_ over four example regions, i.e., Region #1 (**e**), Region #2 (**f**), Region #3 (**g**) and Region #4 (**h**). Region #1 in north Japan (139°E–145°E, 41°N–47°N), Region #2 in western Asia (64°E–70°E, 35°N–41°N), Region #3 in southeastern China (113°E–119°E, 27°N–33°N) and Region #4 in Central America (81°W–87°W, 8°N–14°N). The solid curves in **c**, **d** are fitted with extreme-temperature scatters using the LOWESS method, while the solid lines are fitted by linear regression method (*p* value < 0.05). Dashed lines are C–C scaling

Both fast flow and precipitation extremes exhibit three types of behaviour with temperature (Fig. 2c, d and Supplementary Fig. 10): (i) a monotonic increase with temperature, (ii) a monotonic decrease with temperature or (iii) a hook-like structure^32,33^, where extremes increase with temperature up to a threshold (hereafter called peak point temperature) and then decrease with a warming temperature. As examples, we examine more closely the three typical structures in four sample areas (see Fig. 2b). Region #3 indicates a hook-like structure, and the other three regions indicate a monotonic (increasing or decreasing) scaling structure. To have a better understanding of the scaling robustness, we also present scaling curves, all significant at a 0.05 level, of different stations from the four example regions (see Methods, Supplementary Fig. 11). We attempt to understand the extreme decline characteristics under high temperature by evaluating RH data on wet days (i.e., on days with precipitation over 0.1 mm/d) against temperature (Fig. 2e–h). The change in RH tends to coincide with the scaling relationship, i.e., although Region #1 and Region #2 both indicate monotonically increasing scaling behaviour, Region #1 shows an overall increase in RH with warming and has a super C–C scaling for precipitation–temperature relation, while Region #2 shows a RH decrease and sub-C–C scaling (Fig. 2e, f). The discrepancy over Region #1 and Region #2 implies that RH changes weaken or strengthen the C–C scaling, emphasising the role of atmospheric dynamics in addition to thermodynamics for extremes. The thermodynamics for extremes hinges on the assumption that precipitation intensity should be proportional to changes in the saturation vapour pressure, neglecting moisture limitation and energy constraints^34^. Atmospheric dynamics by affecting large-scale subsidence, advection and atmospheric humidity can also modify precipitation and its extremes in response to a changing climate. For instance, over land regions, RH tends to decrease under high temperatures, and reduced moisture availability could partially account for an offset of precipitation intensities due to increased saturation vapour pressure^35^. Importantly, a hook-like structure of RH as a function of temperature is found in Region #3, where RH begins to decline sharply near the peak point temperature in the scaling of precipitation and fast flow extremes, while the steep RH drop occurring in Region #4 coincides with a negative scaling (Fig. 2g, h). The above conclusions also hold when we evaluate RH data one day prior to rain (Supplementary Fig. 12).

As a metric for precipitation intensity–temperature relationship, C–C scaling is thought to be applicable only when there is no moisture limitation or when RH is fairly steady^36,37^. This may not be the case over land, however, where very warm temperatures may imply a large saturation deficit and increased aridity in the absence of sufficient evaporation or moisture advection^37^. Indeed, RH over land regions is decreasing with global warming compared to the ocean where it is relatively steady^35–37^. This is due to a constraint in nearly equal equivalent potential temperature changes over the ocean and land. As land regions have higher Bowen ratios, this implies higher absolute temperature changes and lower RH over continents^38^. This continental moisture limitation would then inhibit the development of convection and extreme precipitation and result in less intense rainfall and storm runoff response^39^.

The monotonic increase of precipitation extremes with temperature is the dominant phenomenon at high latitudes such as in northern Europe, western Asia, southern Australia and Russia, while most areas over the midlatitudes (e.g., the US, southern Europe, eastern Asia and eastern Australia) typically exhibit a hook-like structure (Fig. 2a). A monotonic decrease is dominant over the tropics, particularly in Southeast Asia, the Indian subcontinent and Central America. Compared with the precipitation–temperature scaling, the hook-like structure of fast flow-temperature scaling prevails over major areas of globe. The negative scaling still dominates the tropical regions except for the Indochina Peninsula (Fig. 2b), where land use change and human activities (Supplementary Figs. 13, 14) have likely impacted the scaling relationships^40–42^. The above findings are robust to the use of different quantiles of extremes, to the same-day versus previous-day local temperature, and to other means of deriving the fast flow extreme (e.g., a baseline of 25th percentile runoff for non-extreme conditions, Supplementary Figs. 15–17).

The negative scaling of extremes at very high temperatures may raise questions about the existence of a potential upper bound for future extremes; however, the decreasing scaling of extremes at high temperatures does not imply such a limit. During extreme precipitation events, the shortwave reflectance of thick cloud, strong surface latent heat fluxes and rain evaporative cooling all contribute to surface cooling, resulting in same-day observation bias towards cooler temperatures. The limitation of the sample size of surface observations at high temperatures might also be an artefact for the occurrence of breakdown in scaling relationships^10,43^. Our work shows that the mean temperatures are colder than the peak point temperature for both precipitation and fast flow over most regions of the globe (Supplementary Fig. 18). Most local temperatures in the regions characterised by a hook-like curve are still described by its ascending branch, suggesting potential intensification of precipitation and runoff extremes with warmer conditions. More importantly, previous work has employed climate models to project that the peak point temperature will increase with warming, shifting the hook curve to warmer temperatures in the future and resulting in a significant increase in precipitation extremes that occur at the peak point temperature^37^.

### Scaling rates of extremes with local temperatures

**Fig. 3:**
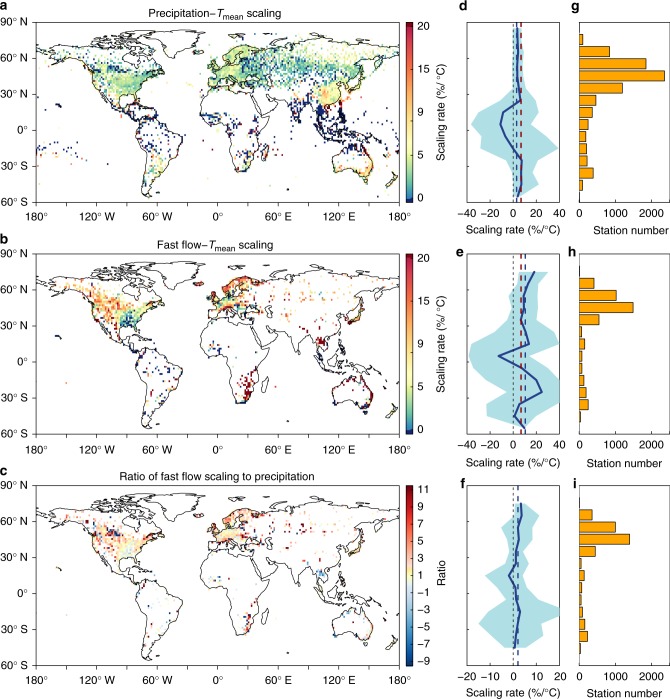
Global scaling results for 99th percentile precipitation and fast flow extremes with local temperature. **a** Scaling results of precipitation extremes with *T*_mean_. **b** Scaling results of fast flow extremes with *T*_mean_. **c** Ratio of fast flow to precipitation scaling with *T*_mean_. **d**–**f** Zonal results based on precipitation scaling, fast flow scaling and ratio, respectively. **g**–**i** Used stations for precipitation scaling, fast flow scaling and ratio analysis, respectively. Solid blue lines indicate the median scaling rate or ratio in each latitude band, and the shading shows the associated 90% confidence intervals. Dashed blue lines indicate the global average values, and dashed red lines in **d**, **e** indicate C–C scaling

To gain further insight into the temperature dependence of extremes, we estimate the spatial distribution of scaling rates of both precipitation–temperature and fast flow-temperature relationships by binning^44–46^; if a hook-like structure was observed, regression fitting was applied only up to the peak point temperature^47–49^. For the precipitation–temperature relationship, a limited region of the globe exhibits a near C–C rate (i.e., between 5 and 9%/°C), dominant over eastern Asia, southeastern Australia, northern Russia, south-western Canada and inner Europe (Fig. 3a). The tropics commonly exhibit negative scaling rates with large scaling variability, from −40%/°C all the way up to 40%/°C (Fig. 3d). This is likely due to the lack of data over the tropics so that the observations do not uniformly sample the conditions there (Fig. 3g). Most regions of the globe indicate sub-C–C rates (i.e., below 5%/°C), such as the eastern US, eastern Europe, southern Russia, southeastern China and Middle Eastern regions. A very super C–C rate (i.e., over 20%/°C) is observed over coastal regions, such as the coastal South China Sea, north-eastern Australia, coastal regions of Africa, and islands (Fig. 3a). Given the importance of oceans in contributing approximately 85% of the moisture to the atmosphere^50^ and the limited role of soil moisture recycling in those regions^51^, the oceans play a dominant role in supplying the moisture needed to generate intense precipitation extremes. In coastal regions, the land–sea breeze is an essential means of moisture advection over land and hence coastal precipitation^52^.

Similarly to precipitation, the negative C–C scaling of fast flow prevails over tropical regions except for the Indochina peninsula (Fig. 3b), this inconsistency may be due to agriculture, human activities and dam construction (Supplementary Figs. 3, 13, 14 and 19), which have affected runoff. Super C–C rates of storm runoff dominate most observed areas of the globe, while very large super C–C scaling prevails over coastal regions (Fig. 3b), such as the western US, southeastern Africa, northern Europe and the coastal regions of Australia, implying that coastal MCSs and land–sea circulations contribute to storm runoff intensification^52,53^. In contrast with precipitation, the sub-C–C scaling rates are mainly observed over the eastern US and over a few limited regions in southern Europe (Fig. 3b), implying that warming climate and anthropogenic changes during the same period, have higher impacts on storm runoff than on daily precipitation extremes. Conducting scaling analysis using *T*_max_ or *T*_min_ in lieu of *T*_mean_, does not alter the conclusions presented here (Supplementary Fig. 20). The runoff-temperature scaling results are globally significant at a 0.05 level, except for a few stations with small scaling rates. The precipitation–temperature scaling is also significant throughout the extra-tropics and only insignificant in regions with low scaling rate (Supplementary Figs. 21, 22). Using local previous-day temperatures produced larger scaling rates than same-day (wet-day) temperatures (Supplementary Fig. 23 and Supplementary Table 1) because of rain evaporative cooling. Rain also reduces same-day temperature via lower surface sensible heat fluxes, so that the previous-day temperature should generally be a better indicator of atmospheric moisture availability.

How does the scaling of fast flow extremes compare to that of precipitation? In the extra-tropics, fast flow usually exhibits higher temperature scaling compared with precipitation, while over certain limited regions in the tropics the fast flow-temperature scaling is opposite that of precipitation–temperature, for example, over the Indochina Peninsula and north-western Australia (Fig. 3c). If we use the 95th percentile extremes or derive the fast flow extreme using other methods such as a baseline of 25th percentile runoff for non-extreme conditions, the above conclusions still hold (Supplementary Figs. 24–26). The runoff without separating base flow has slightly lower temperature-scaling rates than fast flow and the relative scaling with precipitation does not change much (Supplementary Table 1 and Supplementary Fig. 27).

To closely investigate the zonal distribution of scaling rates and ratios, we derive the zonal values in each 10° latitude bin (Fig. 3d–f). The scaling rates for precipitation and fast flow both indicate strong zonal variability, slightly more so for the fast flow. In the tropics, the zonal median scaling rates of precipitation–temperature are almost always below zero and range from −11.1 to 7.4%/°C, while the fast flow-temperature scaling rates show large fluctuations, ranging from −12.6 to 20.1%/°C. Notwithstanding spatial and zonal variation, the zonal median temperature scaling rates for fast flow over the extra-tropics usually falls between 5.4 and 24.8%/°C, which is much larger than that of precipitation scaling (ranging from 3.6 and 7.1%/°C). The zonal median ratios of fast flow scaling rate to precipitation rate are above one at major latitude bands of the globe, although with large variability, especially over the tropics where there are fewer stations (Fig. 3g–i).

The different responses of precipitation and storm runoff to temperature can be attributed not only to warming, but also to factors like land use land cover changes, water and land management and vegetation changes that have altered the underlying surface conditions and hydrological feedbacks and hence storm runoff generation. Fast runoff is generated through infiltration excess and saturation-excess mechanisms, largely impacted by soil condition and storm events in terms of intensity and duration. Rainfall intensifies with warming, until the precipitation intensity becomes larger than the infiltration rate capacity and generates runoff^54^. Such a discrepancy in precipitation and infiltration-excess generated runoff suggests a difference of scaling rate at the ponding point also holds at higher temperatures (Supplementary Fig. 28a, b). Moreover, soil pores fill up sooner with higher-intensity rains, generating more saturation-excess runoff. Those soil condition changes contribute to the nonlinear increase in runoff coefficient (i.e., ratio of excess runoff to total rain) with rain intensity increase (Supplementary Fig. 28b), supported by numerous theoretical considerations and observational studies^54,55^. A larger runoff coefficient suggests a higher scaling rate of runoff than precipitation with temperature (see Methods and Supplementary Fig. 28c).

The hydrologic effects of forest degradation, especially in the tropics, can also increase storm runoff generation^56^. Emerging evidence has been provided for the “infiltration–evapotranspiration trade-off hypothesis”, which states that forest removal reduces the infiltration capacity of soil and the water losses through quick flow are larger than the gains from reduced evapotranspiration^57^. The deforestation impairs the maintenance of base flow, which changes the storm runoff pathway and contributes to larger infiltration-excess runoff yields^58^. All these mechanisms contribute to a stronger storm runoff response to climate and anthropogenic changes than for precipitation.

### Decadal variability of extremes-temperature scaling

**Fig. 4:**
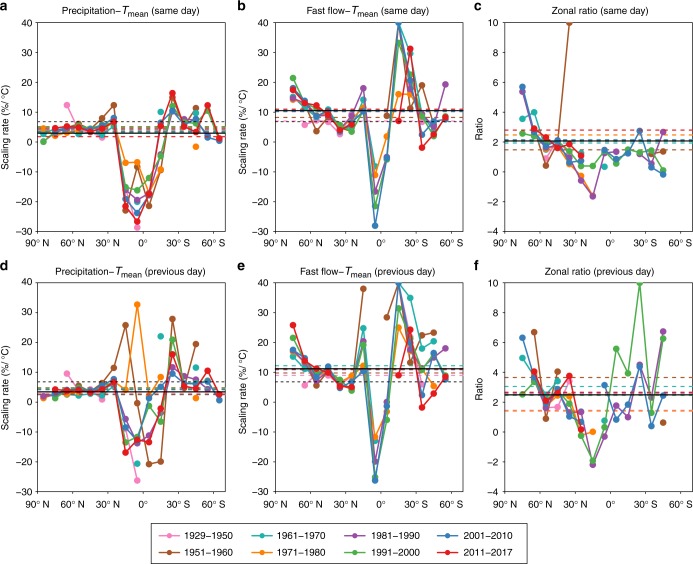
Zonal scaling rates of 99th percentile precipitation and fast flow extremes with local temperature during different periods. **a**, **b** Zonal median scaling results with same-day *T*_mean_ for precipitation extremes (**a**), and fast flow extremes (**b**), respectively. **d**, **e** Zonal median scaling results with previous-day *T*_mean_ for precipitation extremes (**d**), and fast flow extremes (**e**), respectively. **c** Zonal median scaling ratio on same-day *T*_mean_. **f** Zonal median scaling ratio on previous-day *T*_mean_. Dashed colourful lines indicate the global average scaling or ratio results, solid black lines indicate long-term global average results, and dashed black lines in **a**, **b**, **d**, **e** indicate C–C scaling

Despite the strong evidence for climate and anthropogenic influence on storm runoff extremes increase that we have presented so far, it is important to consider the potential confounding effect of decadal variability on these results. We evaluate the influence of decadal variability in the scaling of both precipitation and fast flow extremes, by splitting the total period into eight consecutive time period bins instead of one (Fig. 4a, b, Supplementary Figs. 29, 30). Precipitation and storm runoff scaling in different time period bins show similar zonal characteristics, i.e., tropical regions indicate negative scaling rates with large variability (ranging from −30 to +40%/°C rate) probably due to a lack of stations (Supplementary Fig. 31), while extra-tropic areas mainly show positive scaling rates. Even though it exhibits decadal variability, fast flow mostly exhibits a super C–C scaling whereas precipitation usually exhibits a sub-C–C scaling over the extra-tropics, and the zonal median ratio between these two scaling rates is still almost always greater than one over the extra-tropic region (Fig. 4c). The results for previous-day temperature with extremes show qualitatively similar and quantitatively larger scaling rates. The relative change between precipitation and storm runoff are consistent with the above findings (Fig. 4d–f, Supplementary Figs. 32,  33), implying that the fundamental conclusions that a warming climate has important impacts on extreme storm runoff is robust.

## Discussion

Our work reveals a distinct response of rainfall and runoff extremes to temperature, with the scaling rates of runoff extremes being larger than those of precipitation. These strong responses are determined by the comprehensive impacts of global climate warming and anthropogenic changes. Several previous studies have used detection-and-attribution (D&A) framework to systematically detect the natural variability impact versus human influence on climate and hydrological cycle changes^59–61^. Here, it is challenging to directly attribute and detect changes due to warming versus those due to land-surface modifications given the paucity of data; however, along with the temperature itself, several regional features and anthropogenic activities govern the precipitation pattern and runoff generation. For instance, deforestation, removing the protection provided by natural cover, can lead to soil erosion and disturbance of the ecosystem, thus altering surface roughness, infiltration rates and ultimately fast versus base flow. Decreased roughness leads to reduced evapotranspiration, and increased albedo is a main driver of decrease in moisture flux convergence, both of which potentially decrease precipitation^62^. Deforestation can also increase surface runoff as opposed to reducing base flow: as the forest is degraded, soil water retention capacity is impaired, and base flow is lowered being associated with more rain converted to surface runoff^56–58^. The floodplain wetland is also broadened, contributing to a more rapid runoff yield^62^. Carbon dioxide is another important factor that affects precipitation and runoff generation via plant physiology, as plant stomata generally open less widely under increased carbon dioxide concentration^63^. When stomata open less, transpiration is reduced and soil moisture levels can be higher, which is more favourable for fast runoff generation^64^. Several other anthropogenic land use changes, such as irrigation, agriculture and reservoir construction, can moisten the soil, thus contributing to both precipitation and runoff intensification. All these mechanisms are important in shaping the zonal variation and spatial uncertainty indicated in Fig. 3d–f and Fig. 4 between precipitation/runoff extremes and temperature. Future work could aim at detecting and attributing land-surface changes to storm runoff extremes, given that land use changes can have a key impact on the terrestrial water cycle.

In conclusion, this work provides a first global quantitative assessment of the responses of storm runoff extremes to naturally and anthropogenically driven changes in local temperature and atmospheric moisture content. We systematically compare the different responses of storm runoff and precipitation extremes, and assess the influence of decadal variability in the scaling relations. Our results reveal that storm runoff extremes largely exhibit a super C–C scaling over most measured regions of the globe while precipitation extremes usually indicate a sub-C–C scaling, which are both accompanied by spatial and decadal variability. These strong responses imply that anthropogenic changes and intensification of precipitation extremes have significant impacts on storm runoff events. There is an urgent need to increase societal resilience to both climate change and our changing environment, as our finding that storm runoff extremes are intensifying under warming anthropogenic changes would cause major challenges for existing infrastructure systems.

## Methods

### Observational data

Daily precipitation, near-surface air temperature, dew point temperature and wind speed data for the period 1929–2017 are obtained from the National Climate Data Center GSOD dataset, covering 26,592 stations over the world. Daily runoff data during 1918–2017 are obtained from the GRDC dataset, covering 7237 land-based stations (Supplementary Fig. 1). The GSOD dataset, produced by the National Centers for Environmental Information from hourly weather stations observations contained in the integrated surface hourly data set, is probably the largest publicly available international station data set^65^. Of the various land-based weather station data sets that offer daily summary data, GSOD is the only one that includes weather records necessary to measure a location’s RH^66^, which is an important climate variable measuring moisture availability and the scaling of extremes with temperature^12,36^. We also use daily wind speed data and specific humidity data (from which we calculated daily moisture flux convergence) from NCEP–NCAR reanalysis dataset, covering 1948–2017. Global irrigation data during 2000–2008 are obtained from the Food and Agriculture Organisation of the United Nations. Global dam and reservoir dataset and human population-density data are obtained from Socioeconomic Data & Applications Center (SEDAC) of NASA Earth Observing System Data and Information System.

### Data quality control

Before using the data, we conduct strict quality control. We prescreened obviously wrong temperature and precipitation data firstly, such as negative precipitation or *T*_max_ < *T*_mean_ (or *T*_min_ > *T*_mean_). In a second stage, temperature outliers were identified using standard deviation (*σ*) thresholds as suggested in a previous study^67^. The variance of the station time series was calculated for each day using the surrounding 5 days, and the outliers greater than 4*σ* from the mean are corrected to the derived values from the nearby stations at the same day within 1.0° × 1.0° grid box by the inverse distance weighting interpolation method. Similar to temperature data, we corrected those unrealistic high precipitation values using the maximum precipitation at the same day from the nearby stations within the same 1.0° × 1.0° grid box.

### Regression and trend analysis

We only used stations having at least 80% complete data spanning at least 12 years, and then quantile regression method^68^ is employed to estimate the trend of annual extremes (99th and 95th percentile) for *T*_mean_, *T*_max_, *T*_min_, precipitation and runoff for each station. To draw the results map, we averaged the results for stations into 1.5° × 1.5° longitude–latitude grid box.

### Binning scaling of precipitation extremes with temperature

We investigated the scaling relationship between precipitation and temperature by applying a binning method^44–49^. Only data on wet days with precipitation over 0.1 mm/d were used. At each station, the wet events were stratified based on local temperature, and then all events were divided into 12 bins, and we only used stations having at least 100 data in each bin. The 99th (or 95th) percentile daily precipitation extremes in each bin with a variable width was determined, and the median temperature in each bin was used to represent the local temperature for that bin. The peak point temperature was detected by applying the LOWESS method^47,69^ to the scattering pairs. Finally, an exponential regression was used to relate the extreme precipitation (*P*_1_, *P*_2_) with temperature change (∆*T*):

1 $$P_2 = P_1(1 + 0.01\alpha _P)^{{\mathrm{\Delta }}T}{,}$$

where *α*_*P*_ is the scaling rate at which precipitation extreme change with temperature, and could be estimated by a least squared linear method.

### Binning scaling of runoff extremes with temperature

Before examining the scaling relations between storm runoff extremes and local temperature, we matched the meteorological stations with each runoff station in the same 0.5° × 0.5° grid box, and then the average daily temperature of matched stations was used as local temperature. A similar equation as precipitation–temperature scaling was applied in investigating the extreme runoff (*R*_1_, *R*_2_) with local temperature:

2 $$R_2 = R_1(1 + 0.01\alpha _R)^{{\mathrm{\Delta }}T}{,}$$

where *α*_*R*_ is the scaling rate at which runoff extreme change with temperature. If a hook-like structure was observed in the relationship between precipitation–temperature scaling or runoff-temperature scaling, regression fitting is applied only up to the peak‐point temperature^47–49^.

### Comparing scaling rates of runoff and precipitation extremes

Our finds reveal a higher scaling rate of runoff than precipitation with temperature. To assist in explaining these results, we partially attribute the different responses of extreme events to local surface temperature by runoff coefficient change. The runoff could be expressed as runoff coefficient (*β*_1_, *β*_2_) as^54,55^:

3 $$R_1 = P_1\beta _1,R_2 = P_2\beta _2{.}$$

Substituting Eq. (3) into Eq. (2), we obtain:

4 $$P_2\beta _2 = P_1\beta _1(1 + 0.01\alpha _R)^{{\mathrm{\Delta }}T}{.}$$

Substituting Eq. (1) into Eq. (4), we obtain:

5 $$P_1(1 + 0.01\alpha _P)^{{\mathrm{\Delta }}T}\beta _2 = P_1\beta _1(1 + 0.01\alpha _R)^{{\mathrm{\Delta }}T}{.}$$

Eliminating *P*_1_ and taking the logarithm, the following equation is derived:

6 $${\mathrm{\Delta }}T{\mathrm{log}}\left( {1 + 0.01\alpha _P} \right) + {\mathrm{log}}\left( {\beta _2} \right) = {\mathrm{\Delta }}T{\mathrm{log}}\left( {1 + 0.01\alpha _R} \right) + {\mathrm{log}}\left( {\beta _1} \right){.}$$

Eq. (6) can be rewritten as following:

7 $${\mathrm{\Delta }}T{\mathrm{log}}\left( {1 + 0.01\alpha _R} \right) - {\mathrm{\Delta }}T{\mathrm{log}}\left( {1 + 0.01\alpha _P} \right) = {\mathrm{log}}\left( {\beta _2/\beta _1} \right){.}$$

Since *β*_2_ ≥ *β*_1_ in most regions of the globe^54,55^, then

8 $${\mathrm{log}}\left[ {\left( {1 + 0.01\alpha _R} \right)/\left( {1 + 0.01\alpha _P} \right)} \right] \ge 0{.}$$

Therefore, we could find that *α*_*R*_ ≥ *α*_*P*_.

### The fast flow derivation method

To derive fast flow data from runoff series, we separated base flow based upon the recursive digital filter method^70^, which is commonly used in signal analysis and processing. In order to give a sensitivity analysis of the base flow separating method, we also derived the fast flow extreme using other methods such as a baseline of 25th percentile runoff for non-extreme conditions. The results indicated that two separating approaches have little difference (Figs. 2, 3 and Supplementary Figs. 10, 17,  26), thus verifying the robustness of our method. To link extremes with local temperature, we chose near-surface air temperature on same day or prior to one day with extremes, finding that previous-day temperatures show qualitatively similar and quantitatively larger scaling rates than the same-day temperature (Supplementary Figs. 23–27 and Supplementary Table 1).

### Moisture flux convergence derivation

To explain the precipitation changes, we derive the daily MFC data using wind speed and specific humidity data from NCEP–NCAR reanalysis dataset. By vector identity, horizontal MFC (often referred to as moisture convergence) can be expressed as^71^:

9 $${\mathrm{MFC}} = - \nabla \cdot (q{\mathbf{V}}_h) = - {\mathbf{V}}_h \cdot \nabla q - q\nabla \cdot {\mathbf{V}}_h{,}$$

10 $${\mathrm{MFC}} = \underbrace { - {{u}}\frac{{\partial q}}{{\partial x}} - {{v}}\frac{{\partial q}}{{\partial y}}}_{\begin{array}{*{20}{c}} {{\mathrm{advective}}} \\ {{\mathrm{term}}} \end{array}} - \underbrace {q\left( {\frac{{\partial u}}{{\partial x}} + \frac{{\partial v}}{{\partial y}}} \right)}_{\begin{array}{*{20}{c}} {{\mathrm{convergence}}} \\ {{\mathrm{term}}} \end{array}}{,}$$

where *u* and *v* represent the standard two-dimensional wind components in horizontal surface, and *q* is the specific humidity; ∇ = **i**(∂/∂*x*) + **j**(∂/∂*y*), and **V**_***h***_ = (*u*, *v*). In Eq. (4), the advection term represents the horizontal advection of specific humidity, whereas the convergence term denotes the product of the specific humidity and horizontal mass convergence.

### RH data estimation

We use the daily RH data to explain three typical scaling behaviours. To derive the RH data, we use the daily dew point temperature (*T*_dew_) and daily mean temperature (*T*_mean_). The actual vapour pressure (*e*) and saturated vapour pressure (*e*_sa_) was derived by the Clausius–Clapeyron equation^72^:

11 $$e_{\mathrm{s}}(T) = e_{{\mathrm{s}}0}{\mathrm{exp}}\left( \frac{{L_{\mathrm{v}}}}{{R_{\mathrm{v}}}}\left[\frac{1}{{T_0}} - \frac{1}{T} \right] \right){,}$$

where *T*_0_ and *e*_s0_ are integration constants (273.16 K and 611 Pa, respectively), *L*_v_ and *R*_v_ are latent heat of vaporisation (2.5 × 10^6^ J kg^−1^) and vapour gas constant (461 J kg^−1^ K^−1^) respectively. *e*_s_ indicates the saturated vapour pressure at temperature *T*, and RH = *e*_s_(*T*_dew_)/*e*_s_(*T*_mean_).

## Electronic supplementary material

Supplementary Information

## Data Availability

GSOD data sets are available from the National Climate Data Center website (https://catalog.data.gov/dataset/global-surface-summary-of-the-day-gsod). GRDC data sets are available from the from the Global Runoff Data Centre website (http://www.bafg.de/GRDC/EN/Home/homepage_node.html). NCEP–NCAR reanalysis data are available from the National Oceanic & Atmospheric Administration (NOAA) website (https://www.esrl.noaa.gov/psd/data/gridded/data.ncep.reanalysis.html). The global irrigation data are available from the Food and Agriculture Organisation of the United Nations website (http://www.fao.org/nr/water/aquastat/irrigationmap/index.stm). The global human population-density data are available from the Socioeconomic Data And Applications Center (SEDAC) website (http://sedac.ciesin.columbia.edu/data/set/gpw-v4-population-density-rev10). The GRanD data are available from the SEDAC website (http://sedac.ciesin.columbia.edu/data/set/grand-v1-reservoirs-rev01).
