# Supplementary material for: Large increase in global storm runoff extremes driven by climate and anthropogenic changes
Source: Nat Commun. 2018 Oct 22;9:4389. doi: 10.1038/s41467-018-06765-2 (PMC6197252; doi:10.1038/s41467-018-06765-2)
Supplement: Supplementary file 1 — Supplementary Information [file 41467_2018_6765_MOESM1_ESM.pdf]

SUPPLEMENTARY INFORMATION

Large increase in global storm runoff extremes driven by climate and anthropogenic changes

Yin et al.

Supplementary Figures

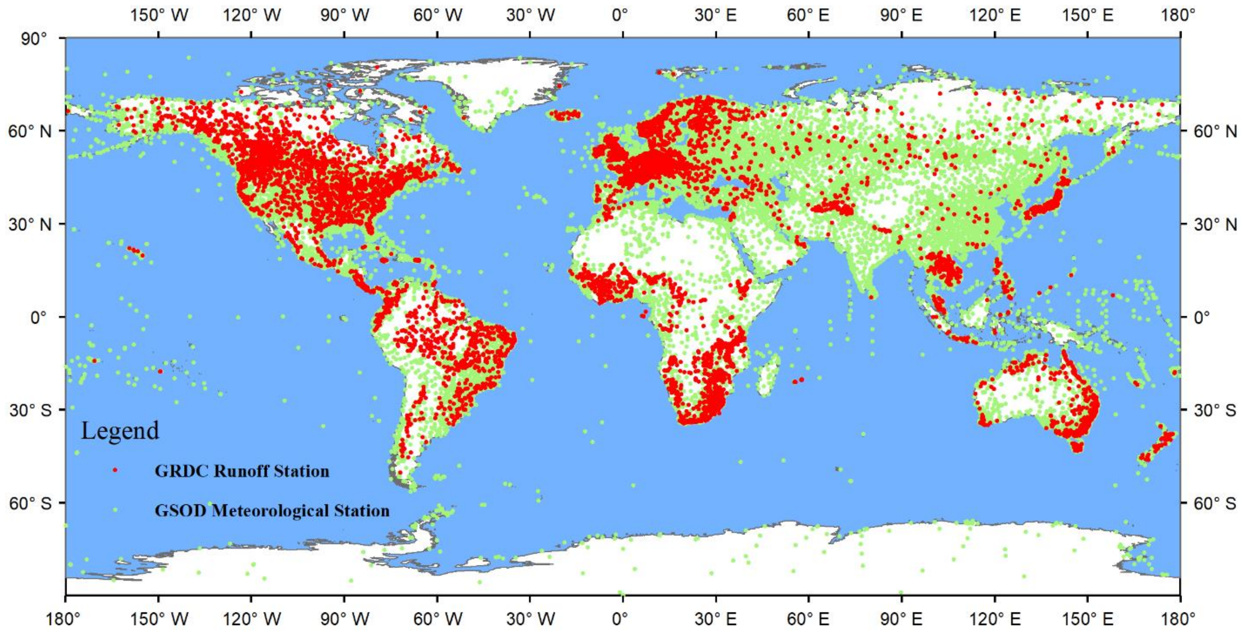

Supplementary Figure 1 | Global daily runoff and meteorological observation stations used in this study. The runoff data are from Global Runoff Data Centre (GRDC) datasets and the meteorological data are from Global Summary of the Day (GSOD) dataset.

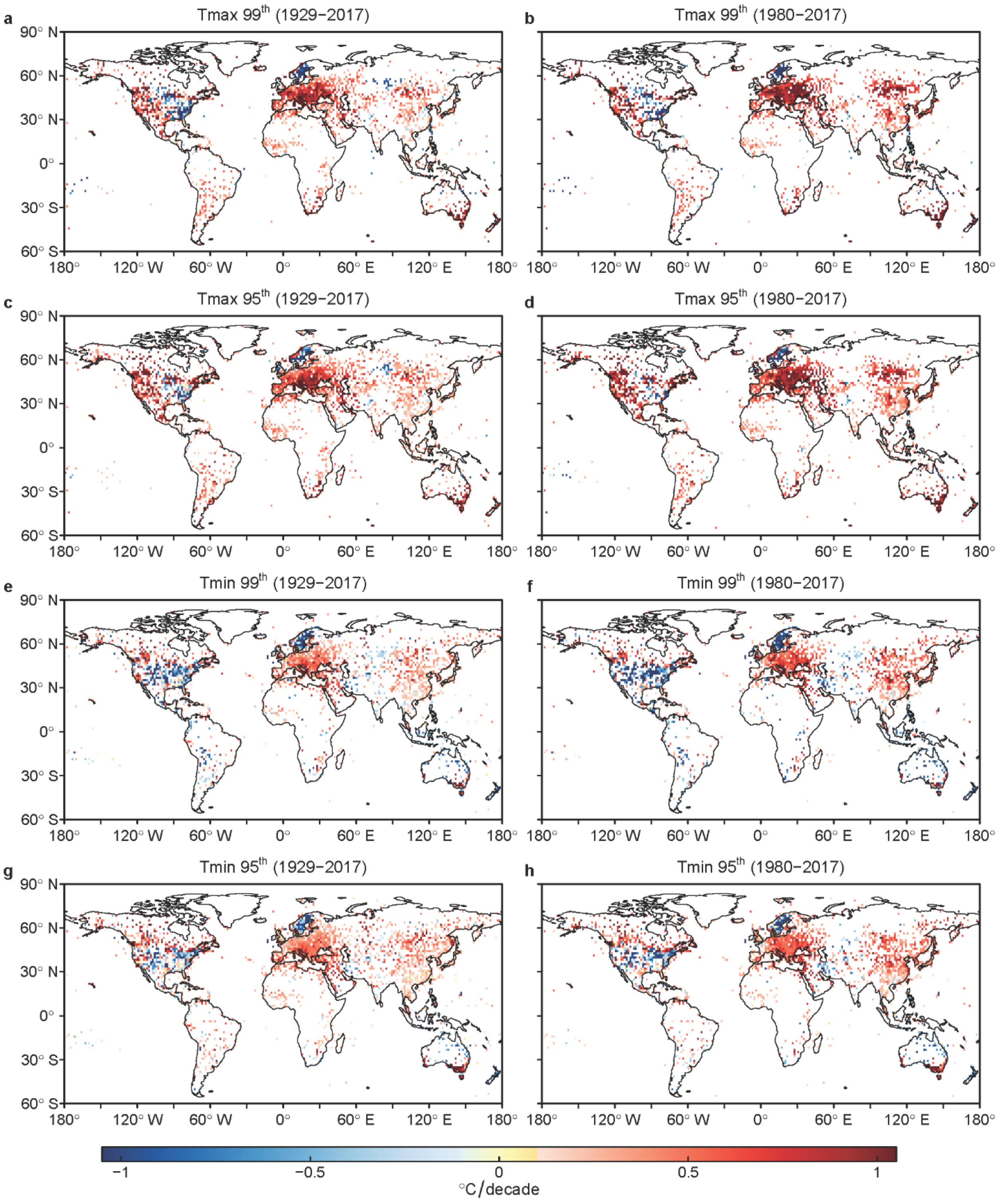

Supplementary Figure 2 | Global trend results for annual daily maximum (Tmax) and daily minimum temperature (Tmin) during two different periods. a-d, trend for 99th percentile Tmax (**a-b**), for 95th percentile Tmax (**c-d**), for 99th percentile Tmin (**e-f**) and for 95th percentile Tmin (**g-h**). White indicates grids with insufficient data or that the trend is insignificant at a 0.05 level.

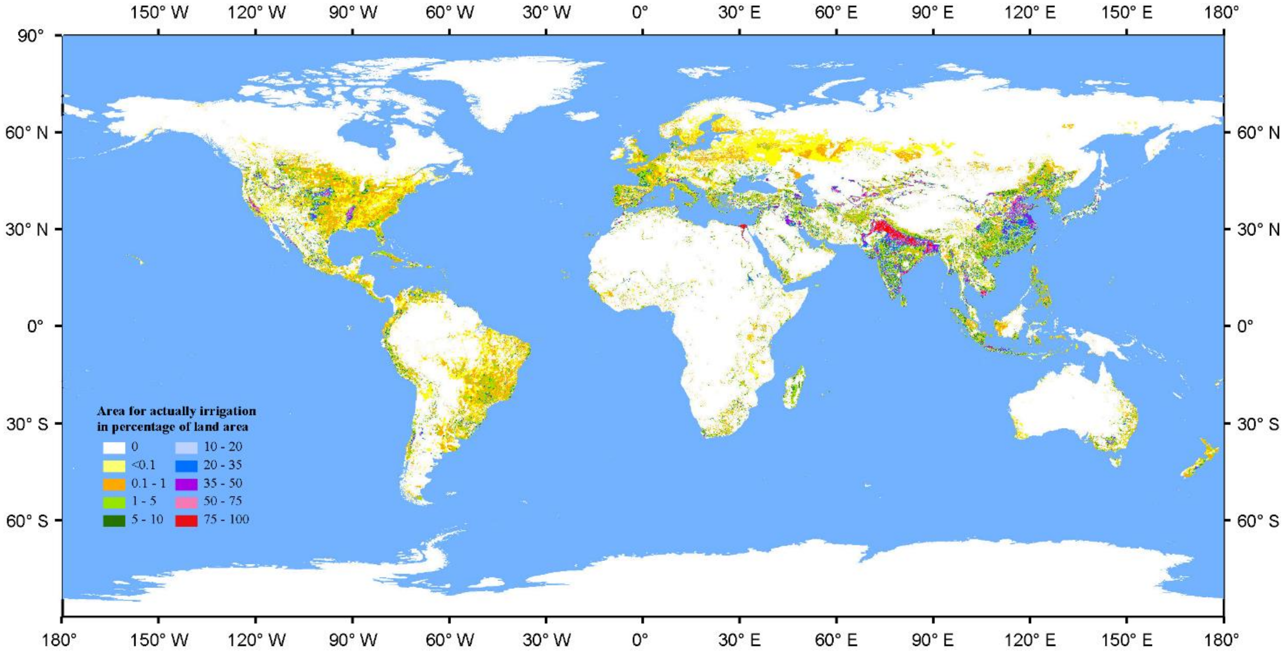

Supplementary Figure 3 | The global map of actually irrigated area in percentage of land area. The base year of statistics is in the period 2000 - 2008 for the majority of countries.

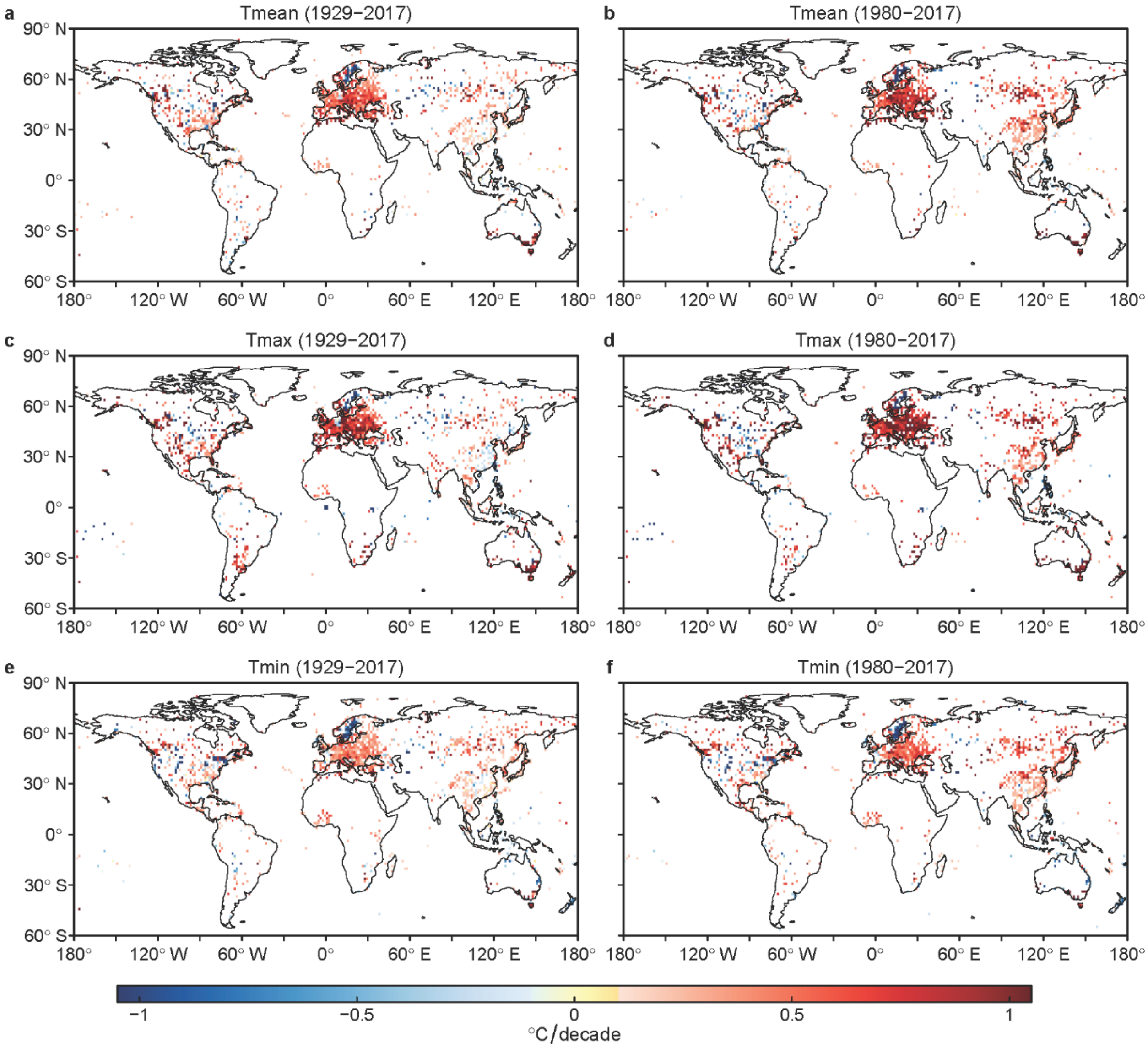

Supplementary Figure 4 | Global trend results for 99th percentile daily temperatures on wet days during two different periods. a-f, trend of Tmean (**a-b**), Tmax (**c-d**), and Tmin (**e-f**), respectively. White indicates grids with insufficient data or that the trend is insignificant at a 0.05 level.

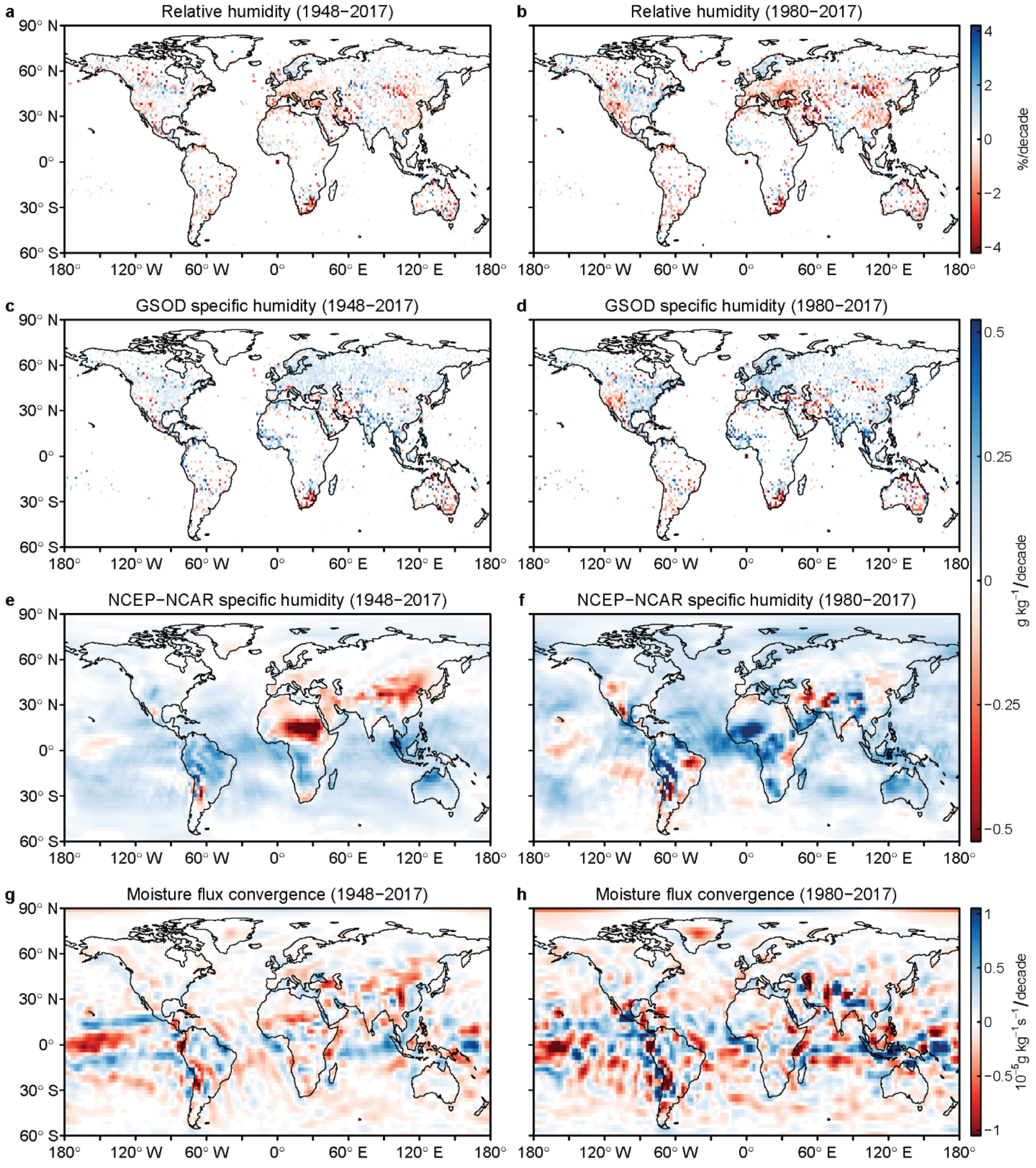

Supplementary Figure 5 | Global trend results for annual mean daily relative humidity, specific humidity and moisture flux convergence during two different periods. a-h, trend of relative humidity (**a-b**), specific humidity of GSOD dataset (**c-d**), specific humidity of NCEP dataset (**e-f**), and moisture flux convergence (**g-h**), respectively.

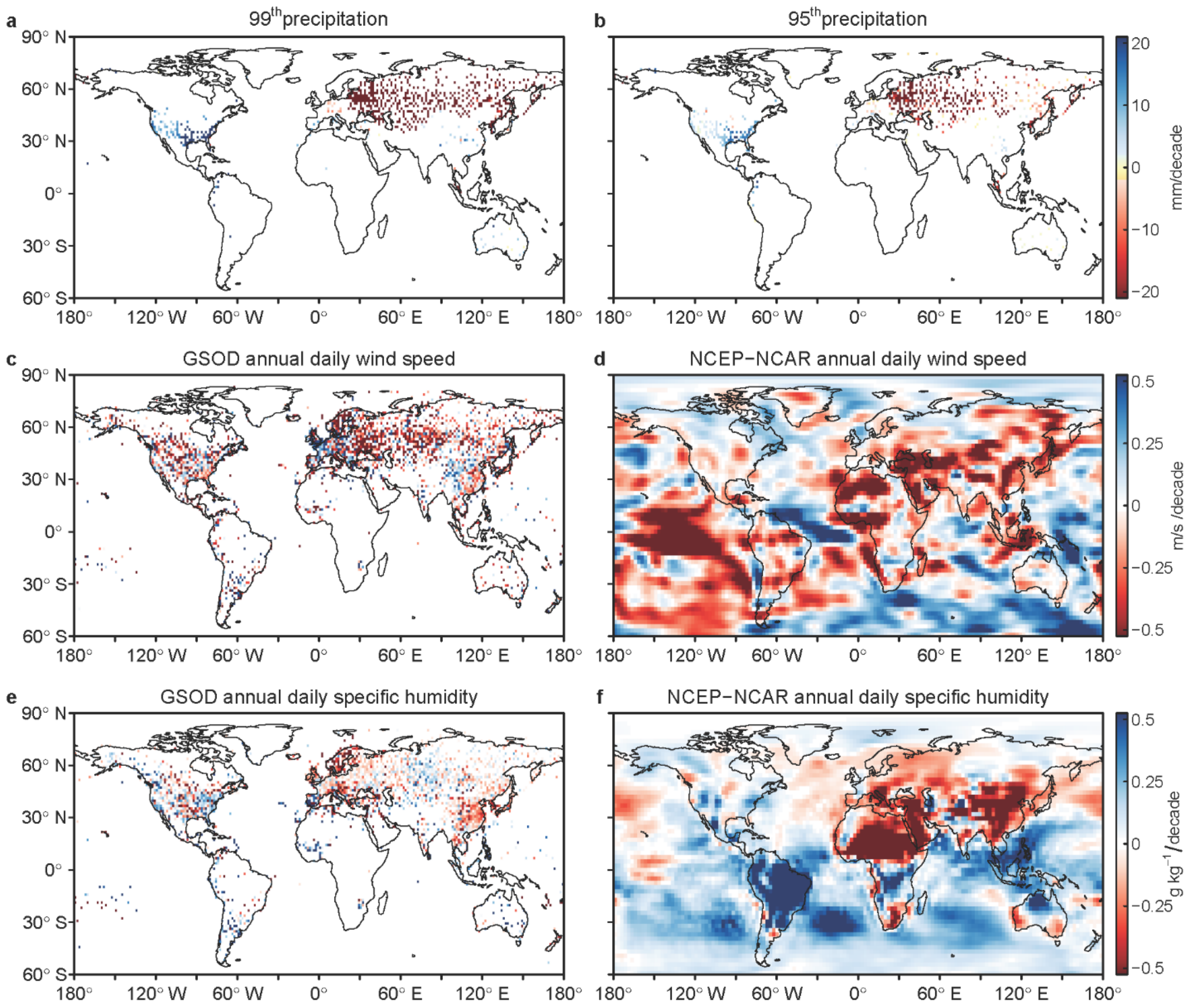

Supplementary Figure 6 | Global trend results for annual precipitation extremes, wind speed and specific humidity during 1961-1980. a-f, precipitation extremes (**a-b**), wind speed (**c-d**), and specific humidity (**e-f**), respectively. White in **a-b** indicates grids with insufficient data or that the trend is insignificant at a 0.05 level.

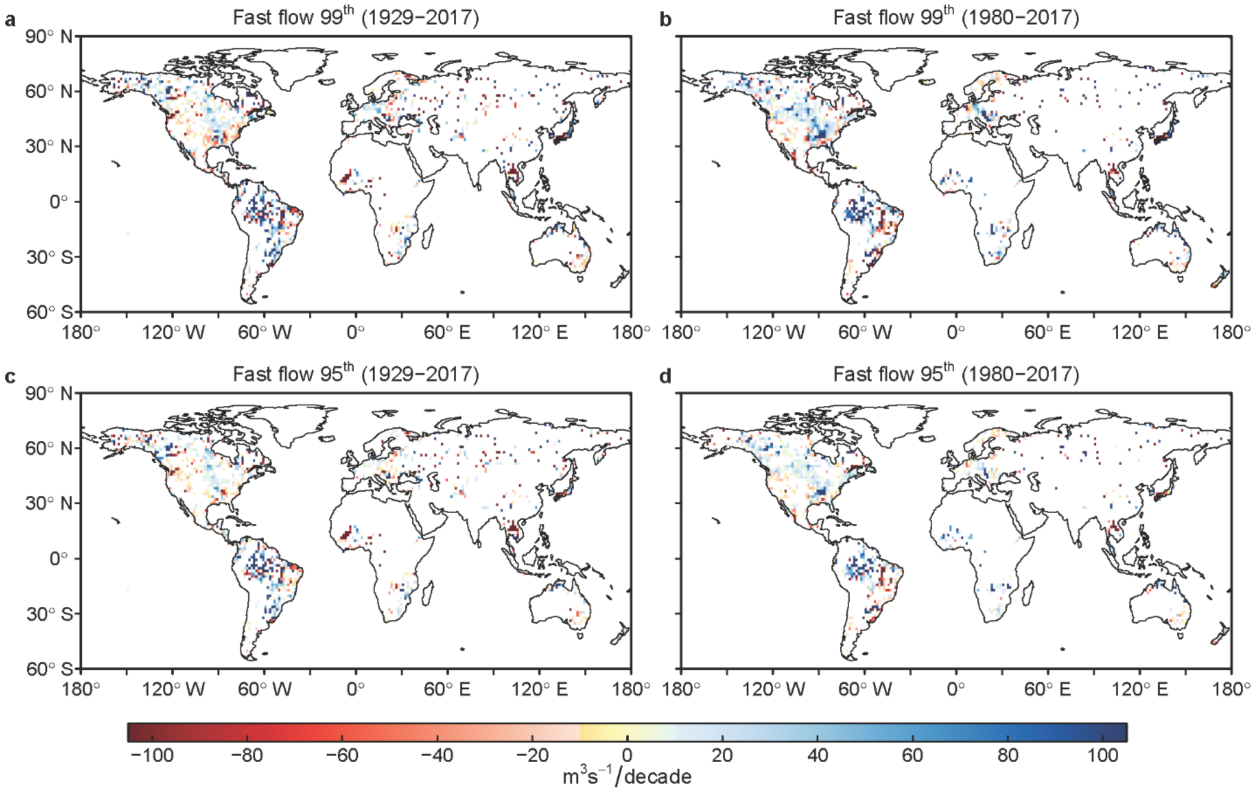

Supplementary Figure 7 | Global trend results of fast flow extremes during two different periods. The fast flow in this map is derived using the recursive digital filter method. **a-d**, trend of 99th percentile fast flow during 1929-2017 (**a**), trend of 99th percentile fast flow during 1980-2017 (**b**), trend of 95th percentile fast flow during 1929-2017 (**c**), and trend of 95th percentile fast flow during 1980-2017 (**d**), respectively. White indicates grids with insufficient data or that the trend is insignificant at a 0.05 level.

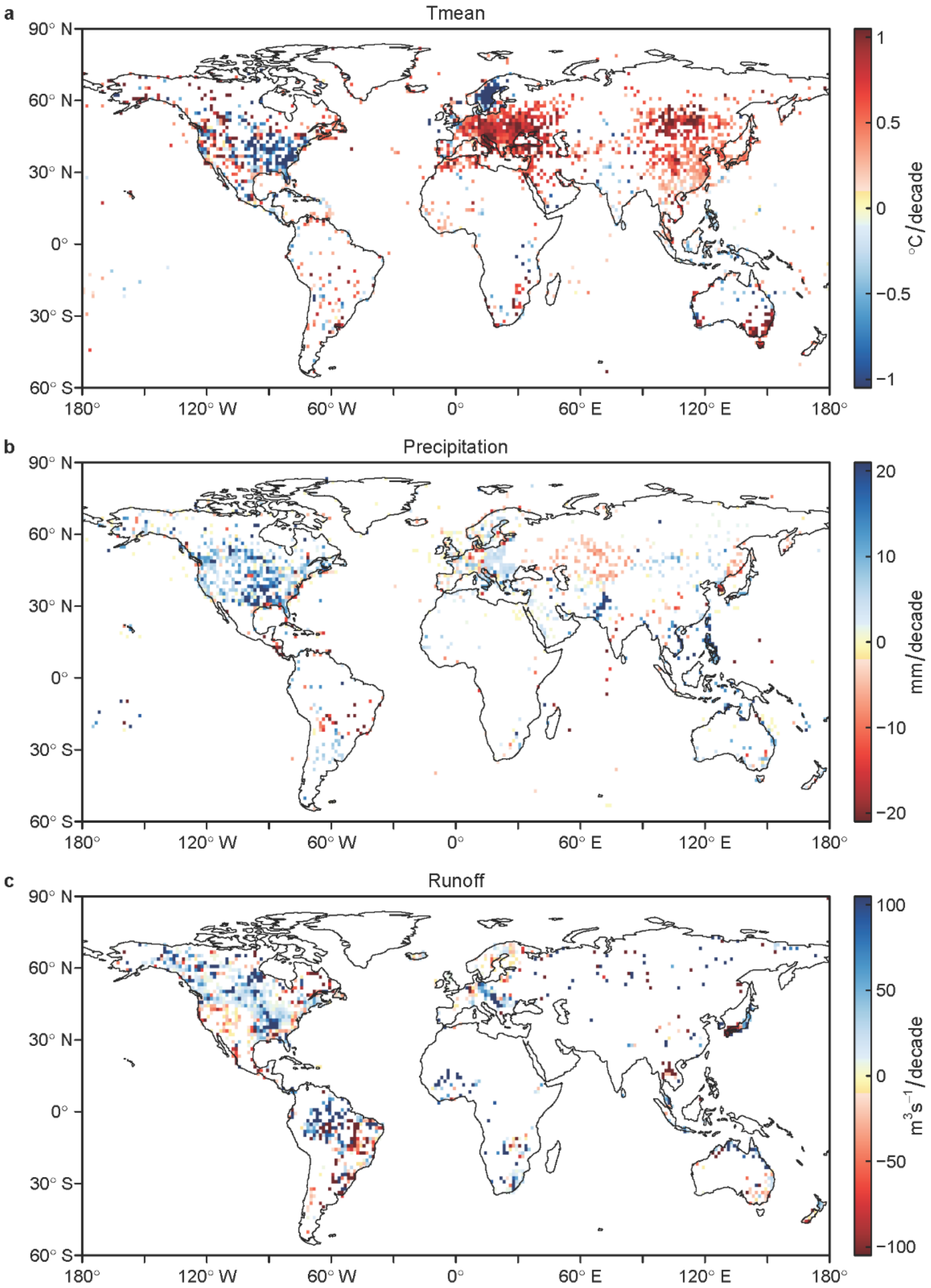

Supplementary Figure 8 | Global trend results for annual 99th percentile daily extremes during 1980-2017. a-c, trend of Tmean (**a**), precipitation (**b**) and runoff (**c**), respectively. White indicates grids with insufficient data or that the trend is insignificant at a 0.05 level.

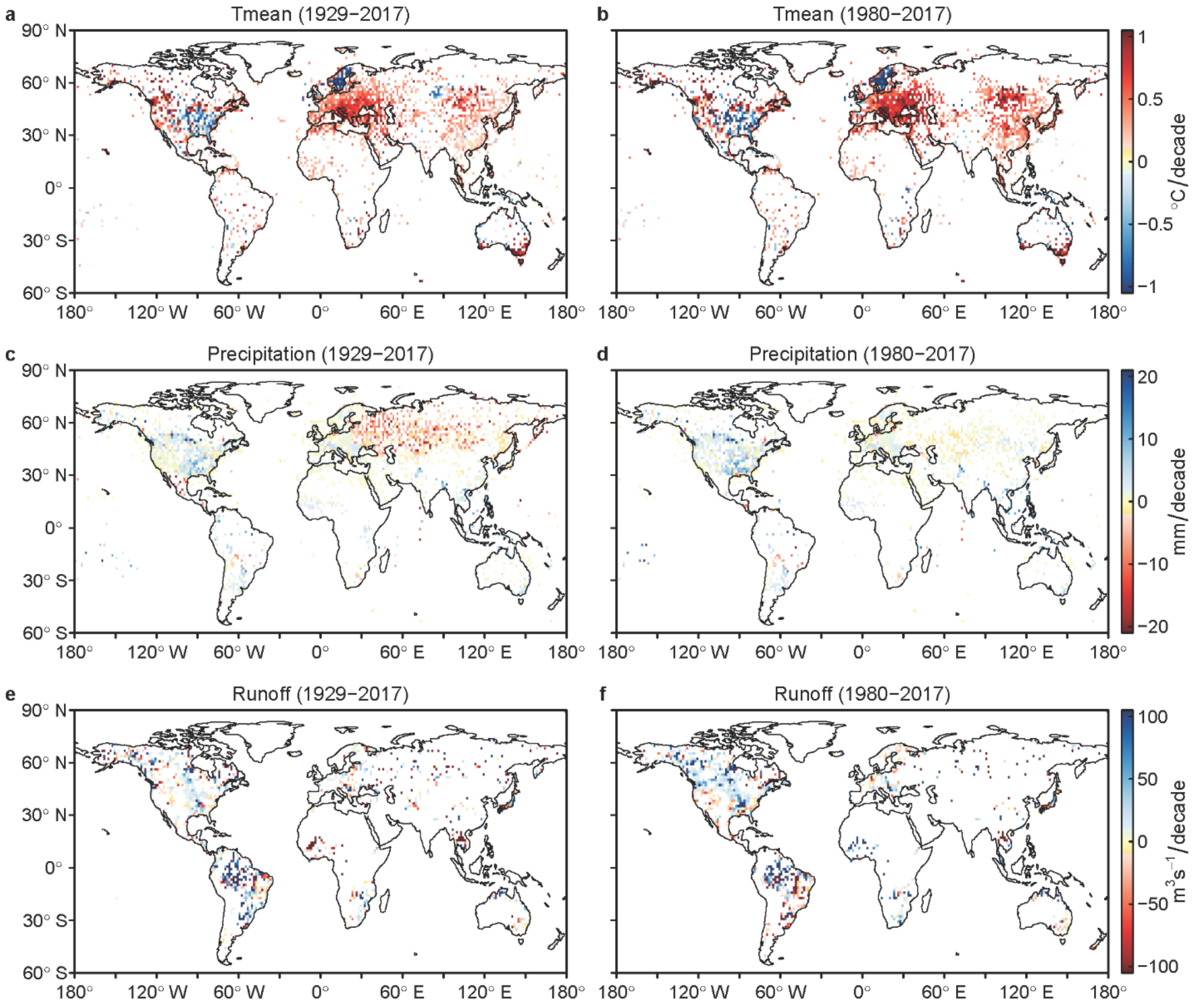

Supplementary Figure 9 | Global trend results for annual 95th percentile daily extremes during two different periods. a-f, trend of Tmean (**a-b**), precipitation (**c-d**), and runoff (**e-f**), respectively. White indicates grids with insufficient data or that the trend is insignificant at a 0.05 level.

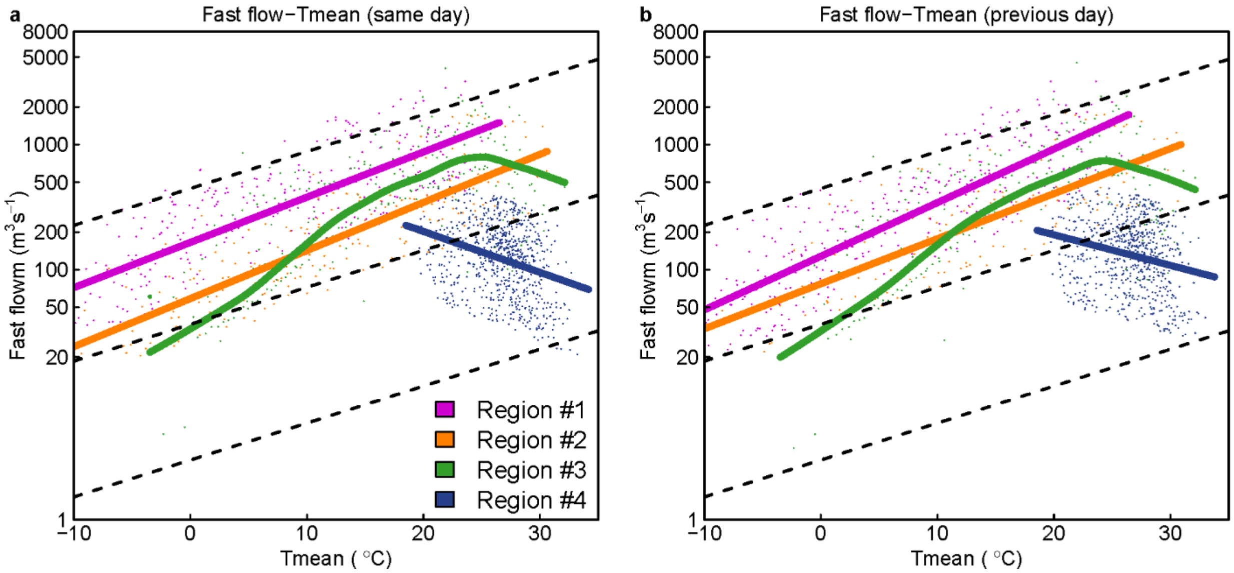

Supplementary Figure 10 | Percentile 99th fast flow extremes varying with Tmean over four example regions. The fast flow in this map is derived by a baseline of 25th percentile runoff for non-extreme conditions in each temperature bin. **a,** relationship between extremes and same-day Tmean. **b,** relationship between extremes and previous-day Tmean. The solid curves are fitted with extreme-temperature scatters using the LOWESS method, while the solid lines are fitted with linear regression method (p-value <0.05). Dashed lines are C-C scaling.

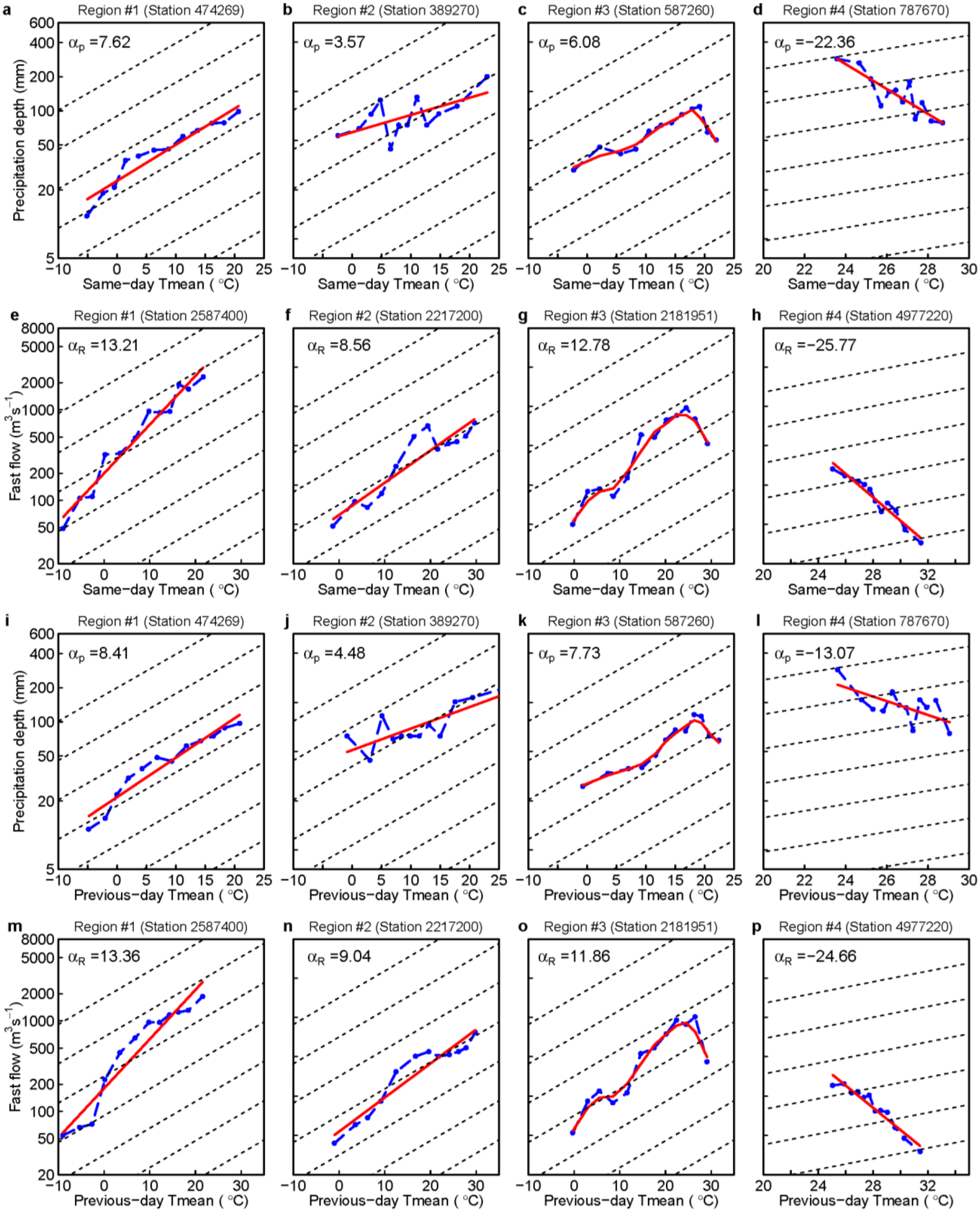

Supplementary Figure 11 | Scaling curves of stations in four example regions. Rainfall station is given as GSOD ID, and runoff station is given as GRDC ID. The upper left value is the scaling rate. Dashed line is C-C scaling, blue scatters are 99th percentile extremes in temperature bins, solid red lines are fitted scaling curve. Region #3 indicates hook structure, and the LOWESS method is used. The other three regions use linear regression, and the results are significant (p-value < 0.05).

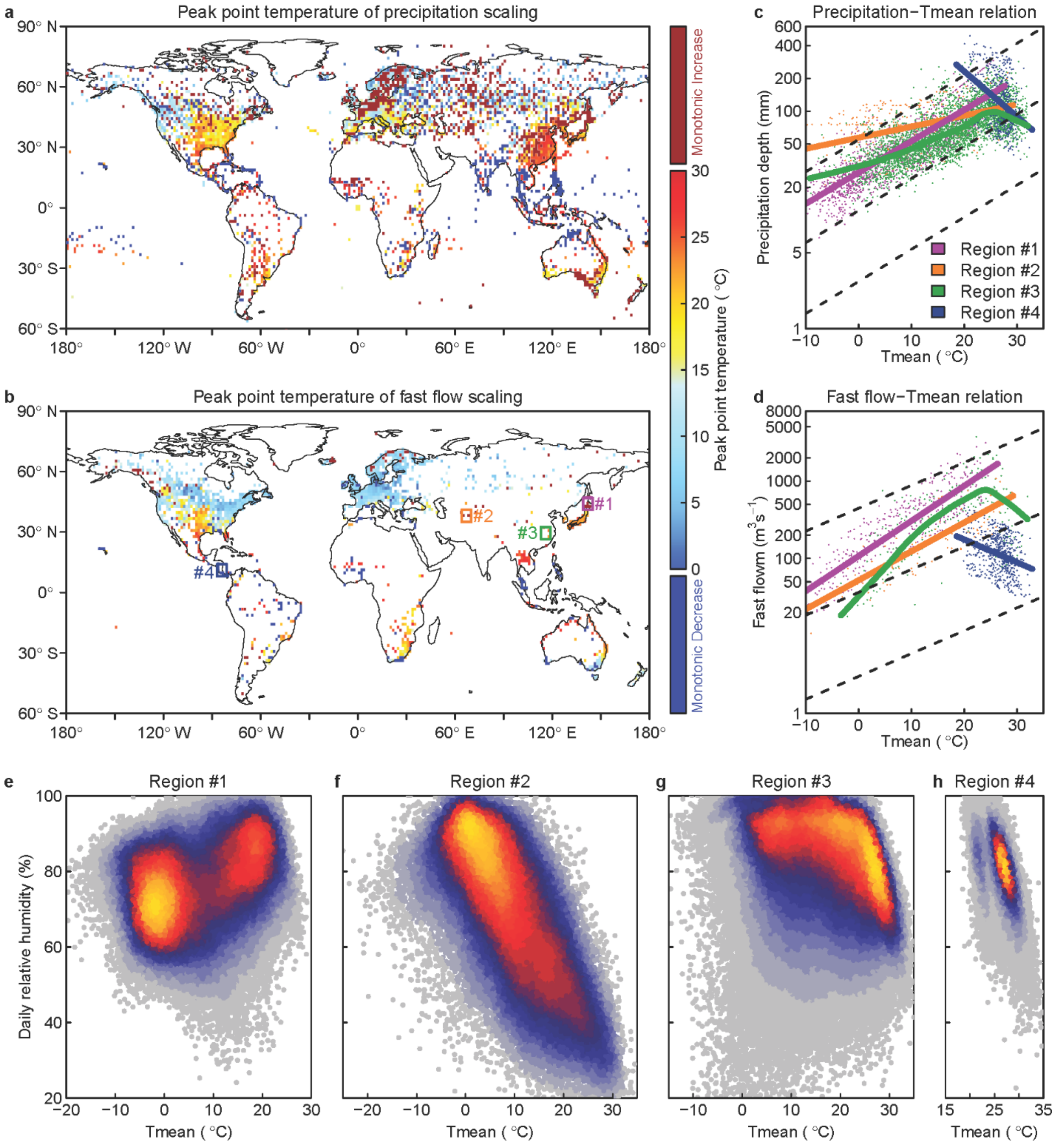

Supplementary Figure 12 | Peak point temperature for percentile 99th extreme with previous-day Tmean and extremes and relative humidity varying with previous-day Tmean over four example regions. a-b, peak point temperature of precipitation extremes (**a**), and fast flow extremes (**b**). **c-d,** relationship between previous-day Tmean with precipitation extremes (**c**), and fast flow extremes (**d**). **e-h**, relative humidity on previous day varying with Tmean over four example regions, i.e., Region #1 (**e**), Region #2 (**f**), Region #3 (**g**) and Region #4 (**h**). The solid curves are fitted with extreme-temperature scatters using the LOWESS method, while the solid lines are fitted by linear regression method (*p*-value <0.05). Dashed lines are C-C scaling.

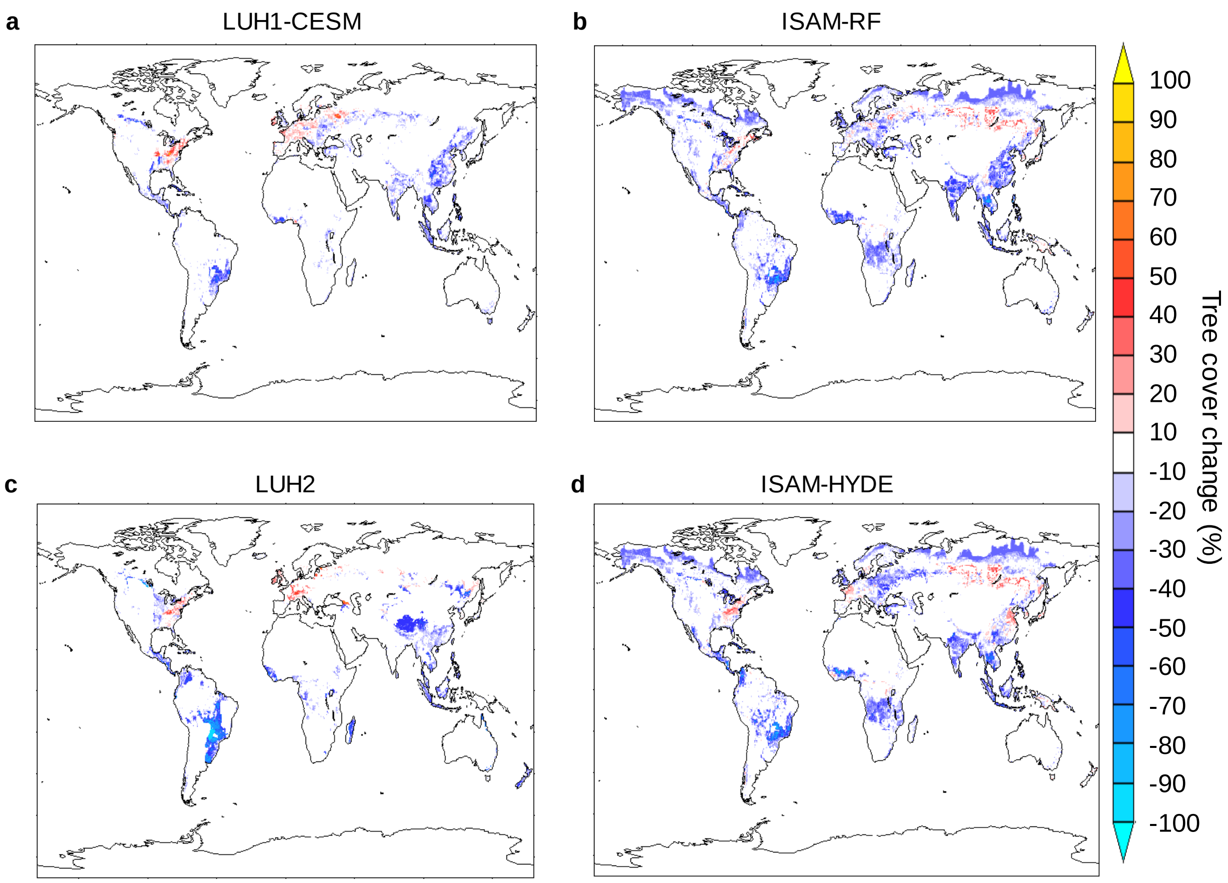

Supplementary Figure 13 | Global map of land use and land cover change during 1901-2005. a-d, change results are analysed from LUHI-CESM dataset (**a**), from ISAM-RF dataset (**b**), from LUH2 dataset (**c**) and from ISAM-HYDE dataset (**d**).

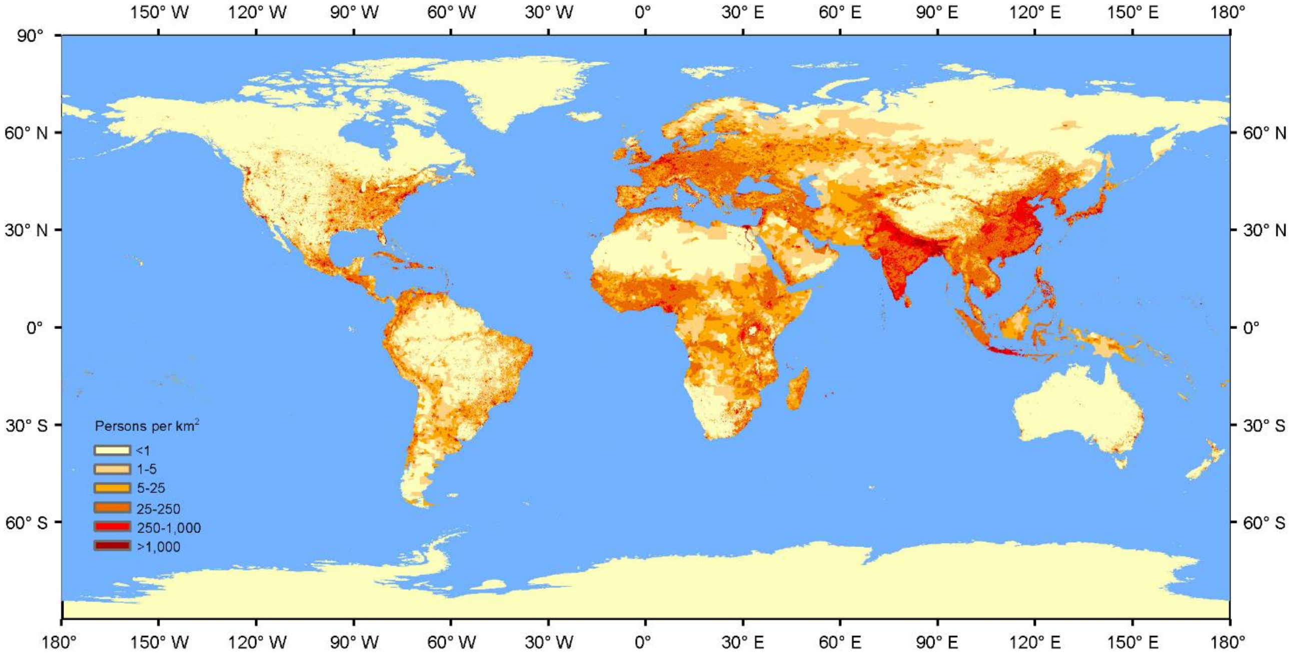

Supplementary Figure 14 | The global gridded human population density. The population count raster is divided by the land area raster to produce population density raster with pixel values representing persons per square kilometres. The data is collected from Gridded Population of the World Version 4 (GPWv4) for year of 2015.

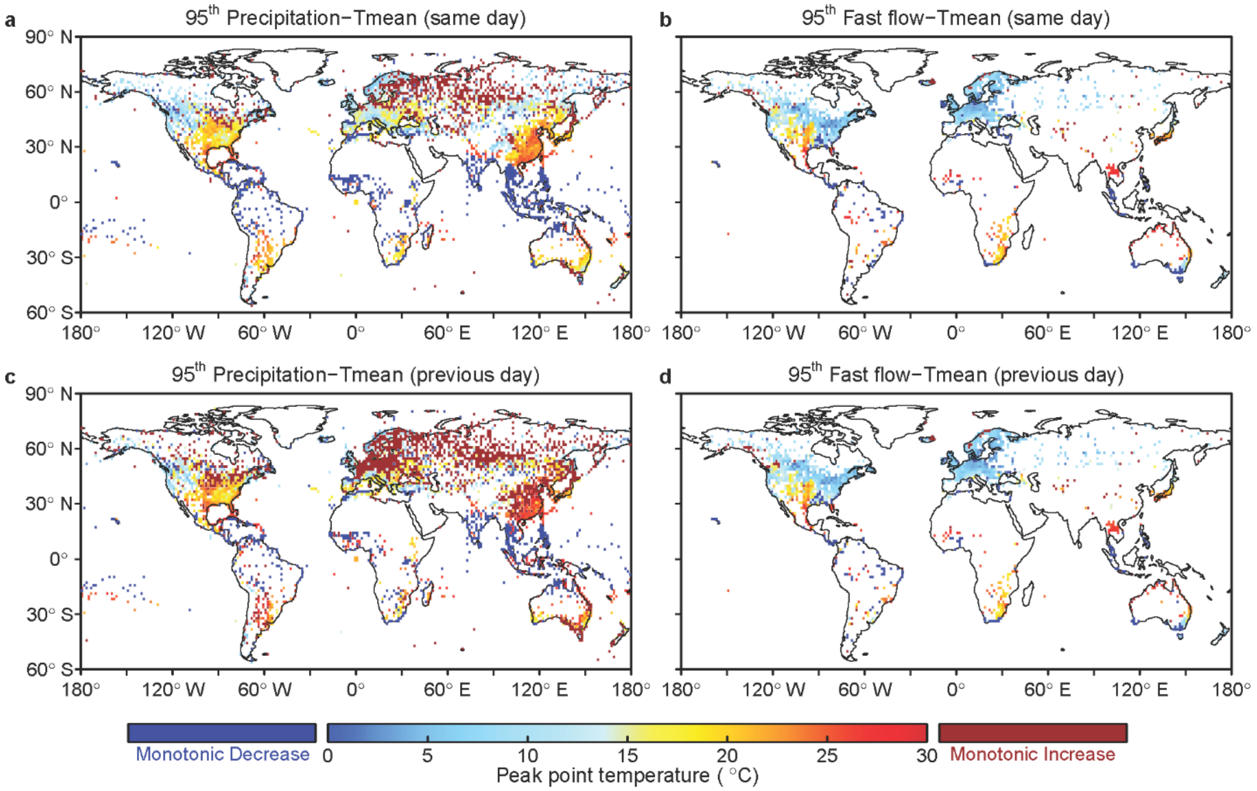

Supplementary Figure 15 | Peak point temperature for percentile 95th extreme with same-day or previous-day Tmean. a-d, 95th precipitation with same-day Tmean (**a**), 95th fast flow with same-day Tmean (**b**), 95th precipitation with previous-day Tmean (**c**), and 95th fast flow with previous-day Tmean (**d**).

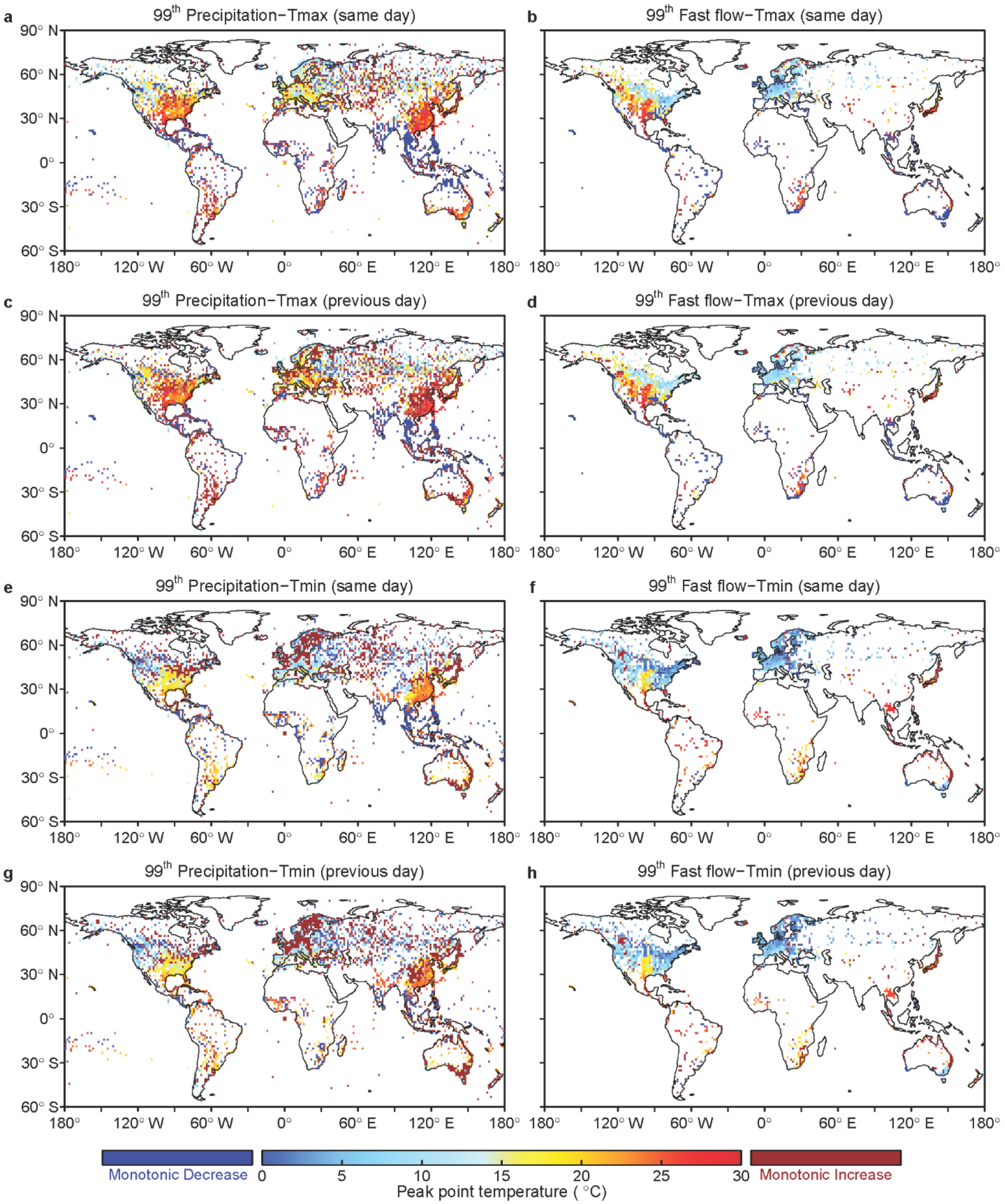

Supplementary Figure 16 | Peak point temperature results for 99th percentile extremes with Tmax and Tmin. a, 99th Precipitation with same-day Tmax. **b**, 99th Fast flow with same-day Tmax. **c,** 99th Precipitation with previous-day Tmax. **d**, 99th Fast flow with previous-day Tmax. **e,** 99th Precipitation with same-day Tmin. **f**, 99th Fast flow with same-day Tmin. **g,** 99th Precipitation with previous-day Tmin. **h**, 99th Fast flow with previous-day Tmin.

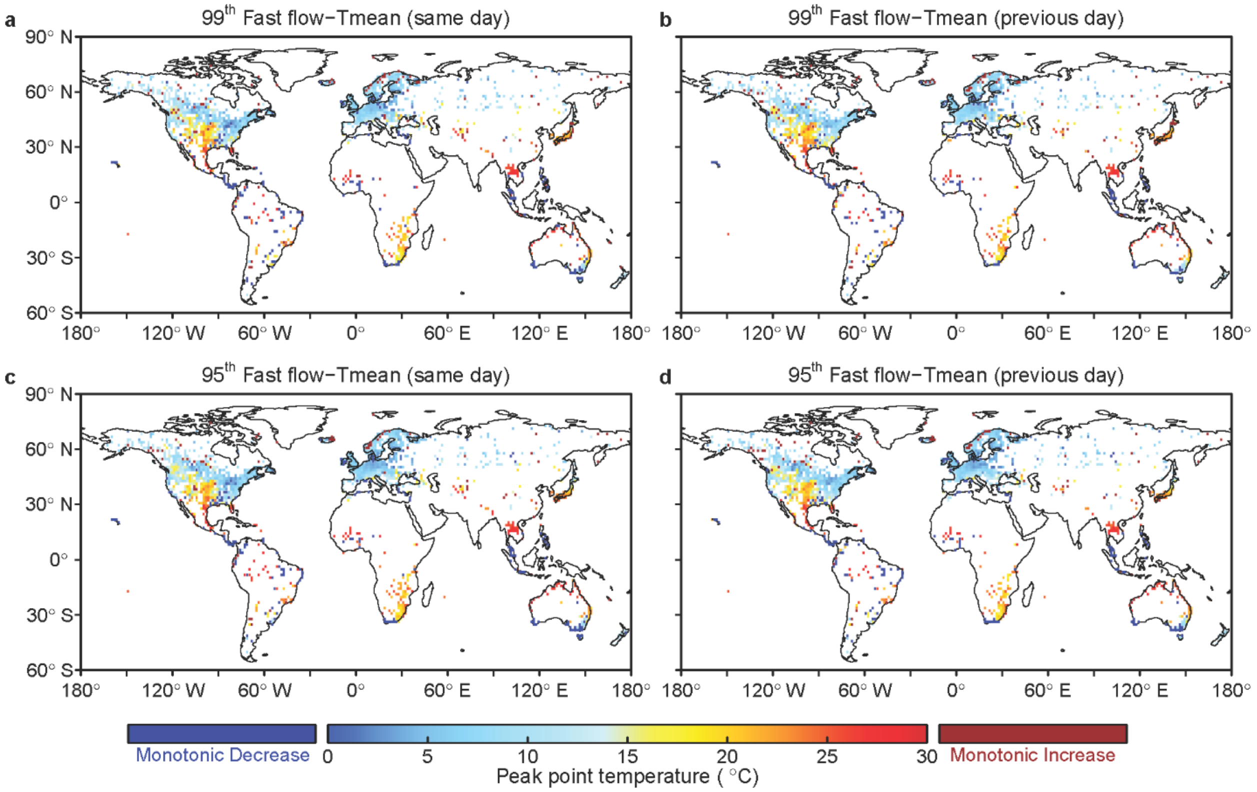

Supplementary Figure 17 | Peak point temperature for fast flow extremes with Tmean. The fast flow in this map is derived by a baseline of 25th percentile runoff for non-extreme conditions in each temperature bin. **a-d,** 99th percentile extremes with same-day Tmean (**a**), 99th percentile extremes with previous-day Tmean (**b**), 95th percentile extremes with same-day Tmean (**c**), and 95th percentile extremes with previous-day Tmean (**d**).

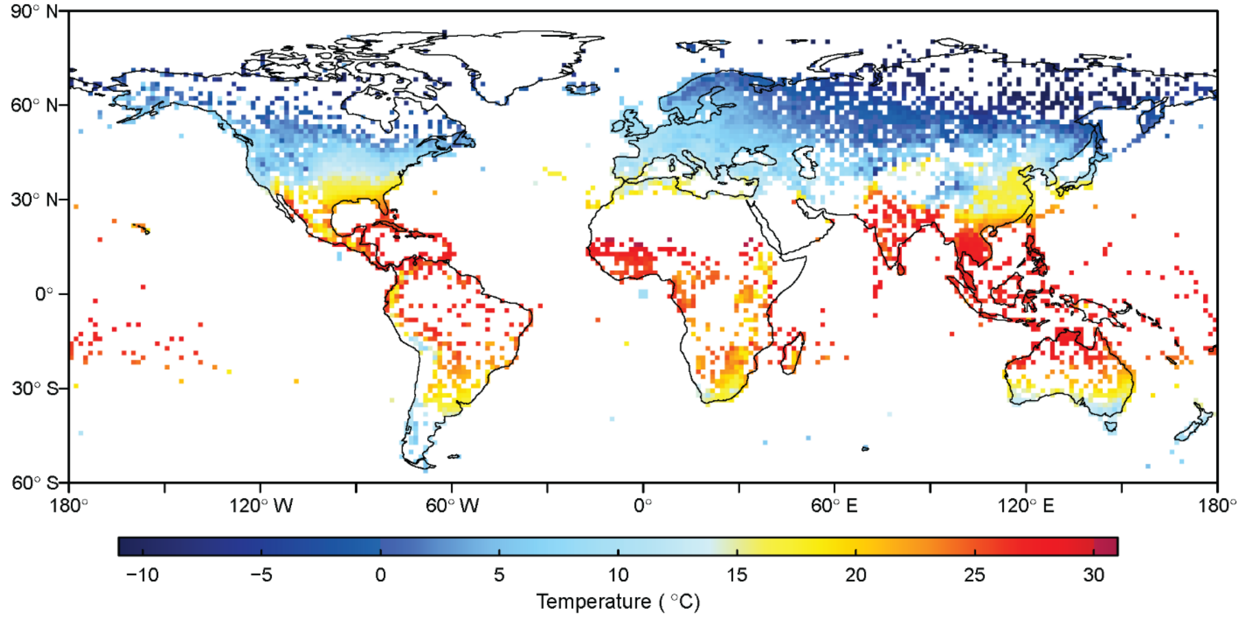

Supplementary Figure 18 | Global mean daily temperature during 1929-2017.

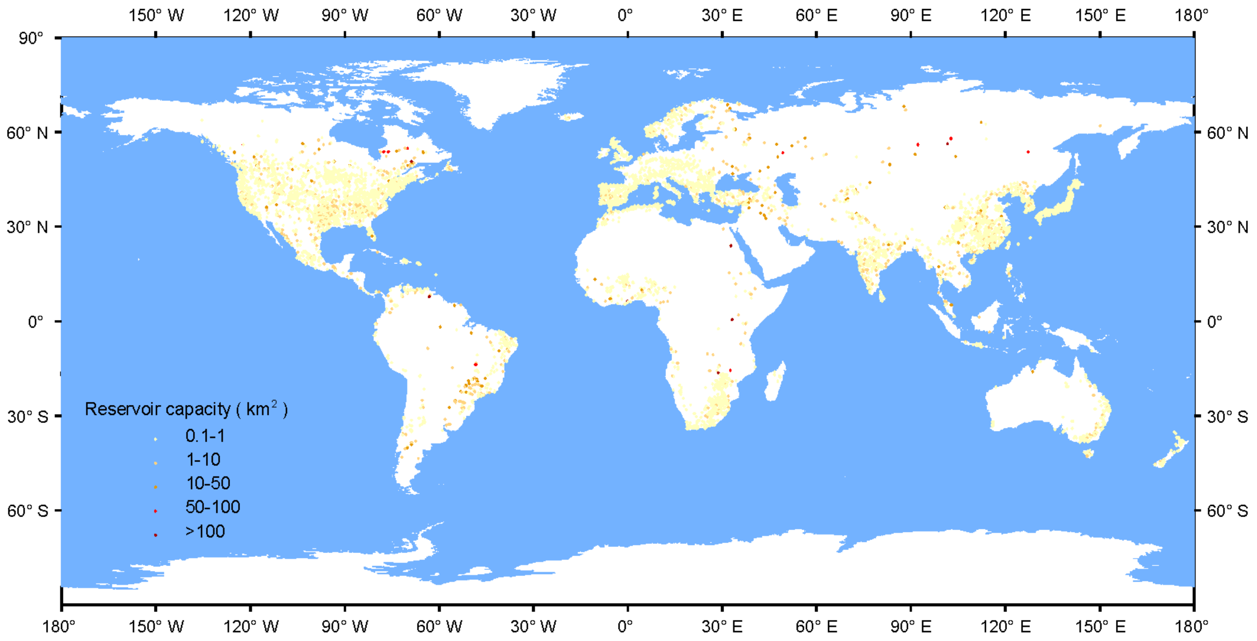

Supplementary Figure 19 | Global distribution of large dams. The data is collected from Global Reservoir and Dam (GRanD) database. This dataset published in 2011 contains 6,862 records of reservoirs and their associated dams.

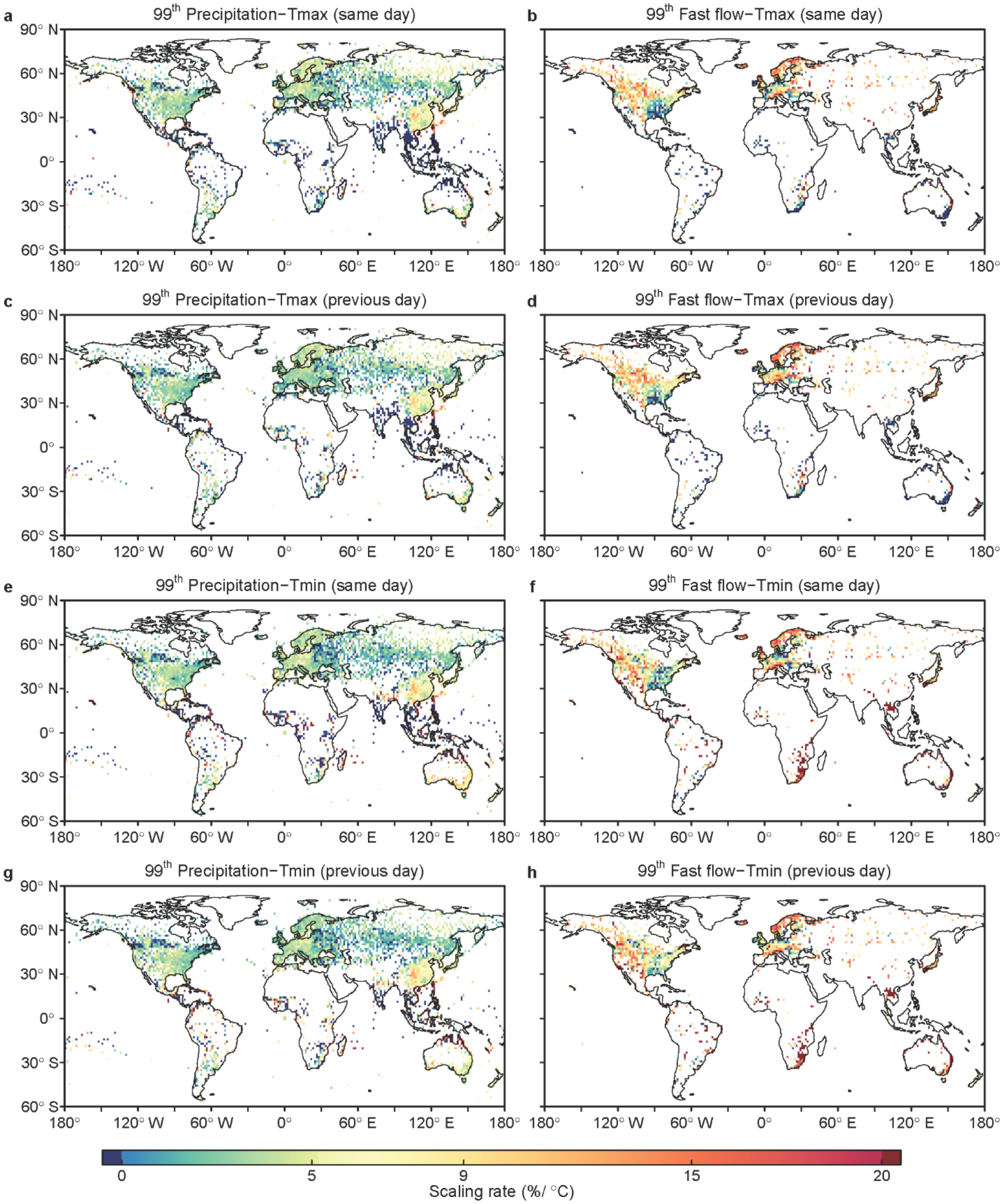

Supplementary Figure 20 | Global scaling results for 99th percentile extremes with Tmax and Tmin. a, 99th Precipitation with same-day Tmax. b, 99th Fast flow with same-day Tmax. c, 99th Precipitation with previous-day Tmax. d, 99th Fast flow with previous-day Tmax. e, 99th Precipitation with same-day Tmin. f, 99th Fast flow with same-day Tmin. g, 99th Precipitation with previous-day Tmin. h, 99th Fast flow with previous-day Tmin.

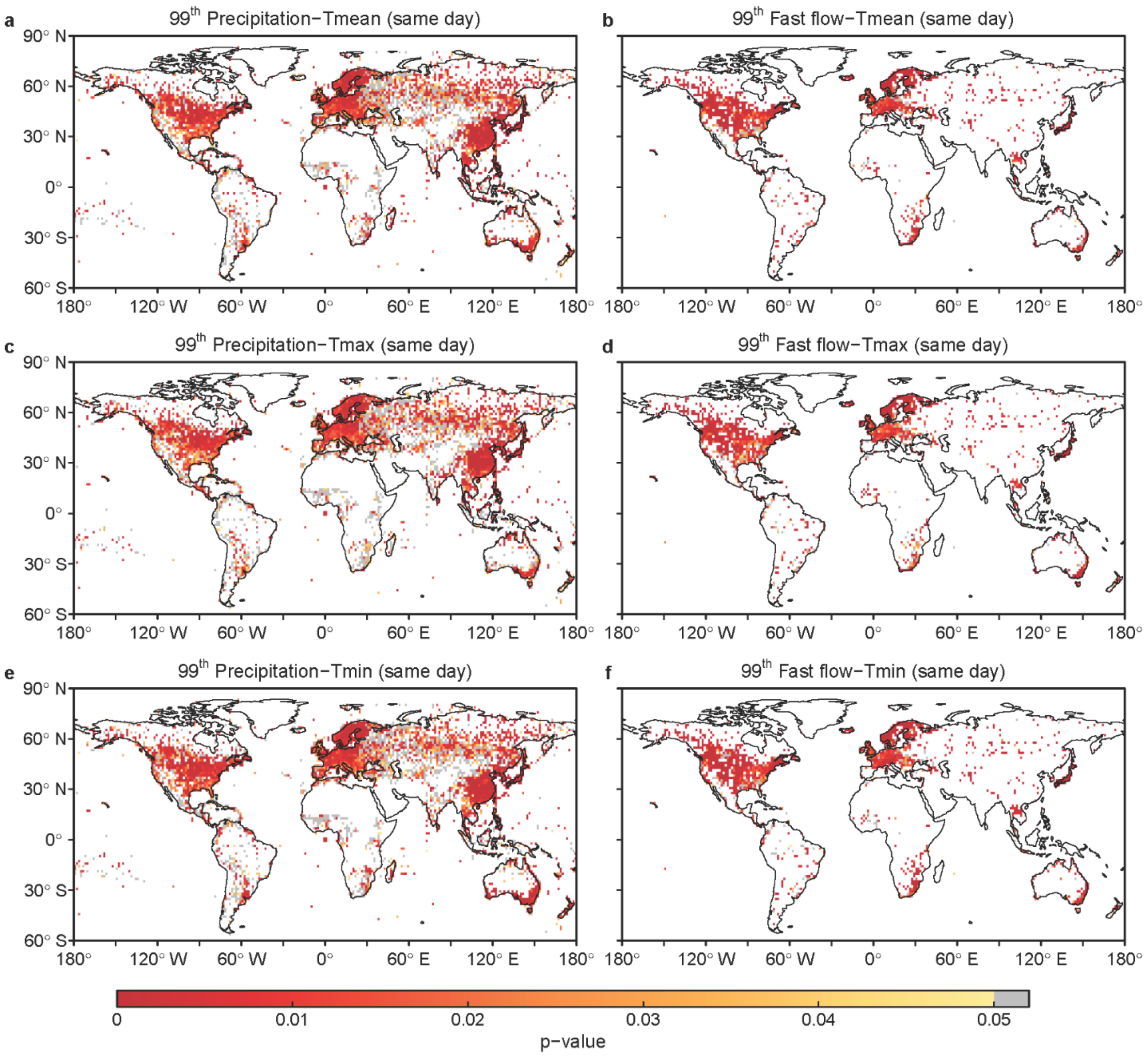

Supplementary Figure 21 | Significance test results for 99th percentile extremes with same-day local surface temperature scaling. a, 99th Precipitation with same-day Tmean. **b**, 99th Fast flow with same-day Tmean. **c,** 99th Precipitation with same-day Tmax. **d**, 99th Fast flow with same-day Tmax. **e,** 99th Precipitation with same-day Tmin. **f**, 99th Fast flow with same-day Tmin.

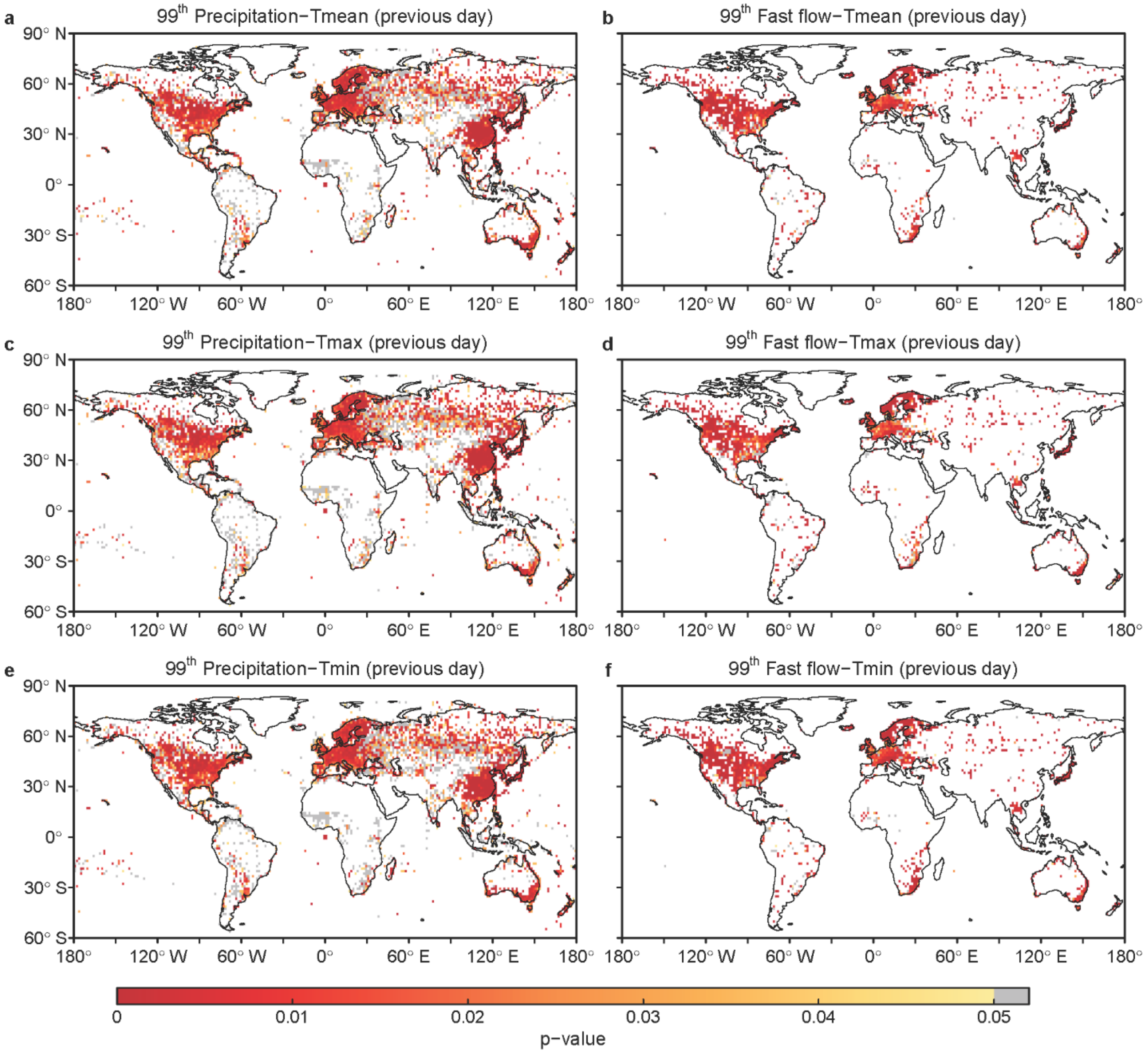

Supplementary Figure 22 | Significance test results for 99th percentile extremes with previous-day local surface temperature scaling. a, 99th Precipitation with previous-day Tmean. **b**, 99th Fast flow with previous-day Tmean. **c,** 99th Precipitation with previous-day Tmax. **d**, 99th Fast flow with previous-day Tmax. **e,** 99th Precipitation with previous-day Tmin. **f**, 99th Fast flow with previous-day Tmin.

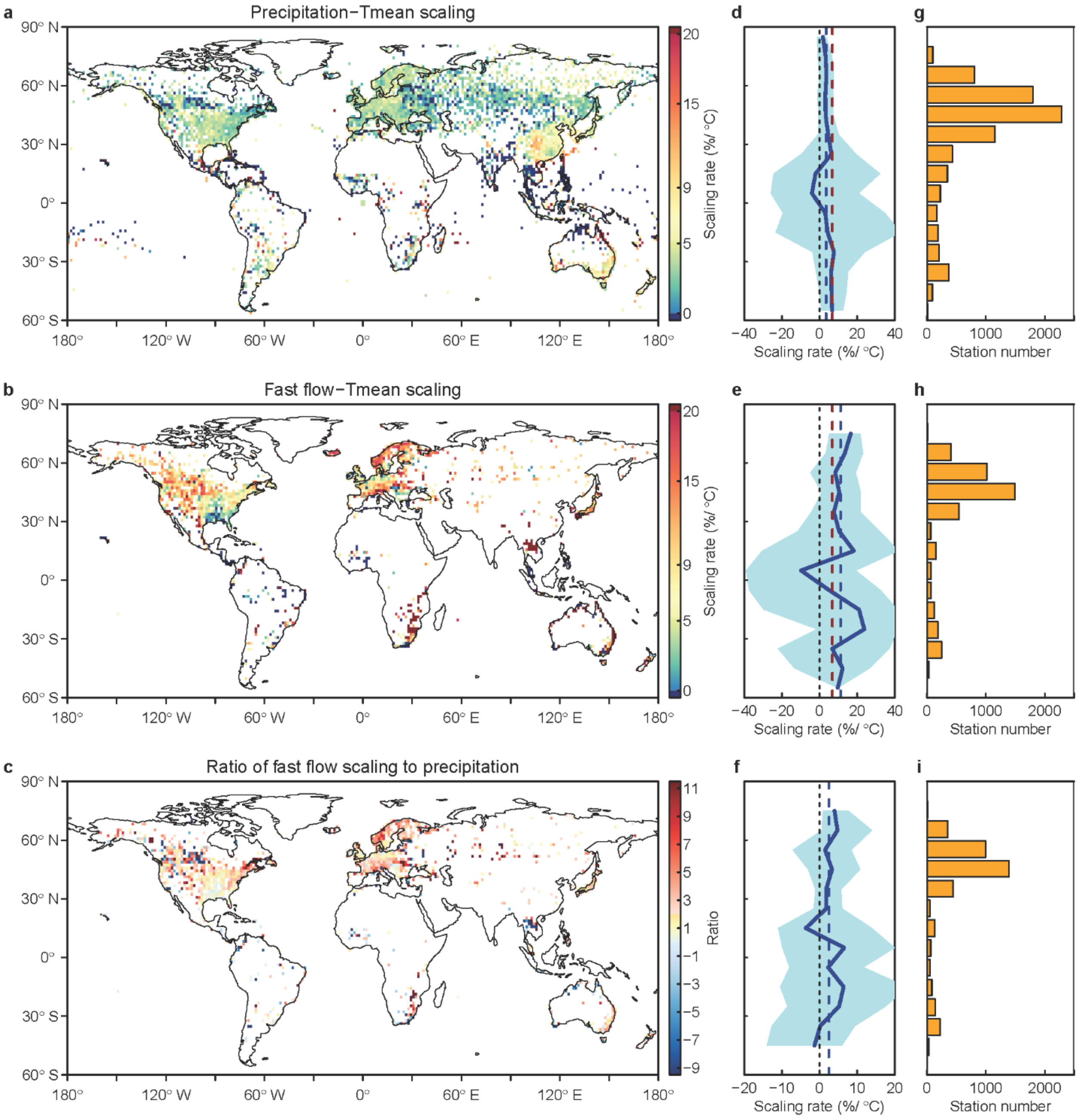

Supplementary Figure 23 | Global scaling results for 99th percentile precipitation and fast flow extremes with previous-day Tmean. a, Scaling results of precipitation extremes with previous-day Tmean. **b**, Scaling results of fast flow extremes with previous-day Tmean. **c,** Ratio of fast flow to precipitation scaling with previous-day Tmean. **d-f**, Zonal results based on precipitation scaling, fast flow scaling and ratio, respectively. **g-i**, Used stations for precipitation scaling, fast flow scaling and ratio, respectively. Solid blue lines indicate the median scaling rate or ratio in each latitude band, and the shading shows the associated 90% confidence intervals. Dashed blue lines indicate the global average values, and dashed red lines in **d-e** indicate C-C scaling.

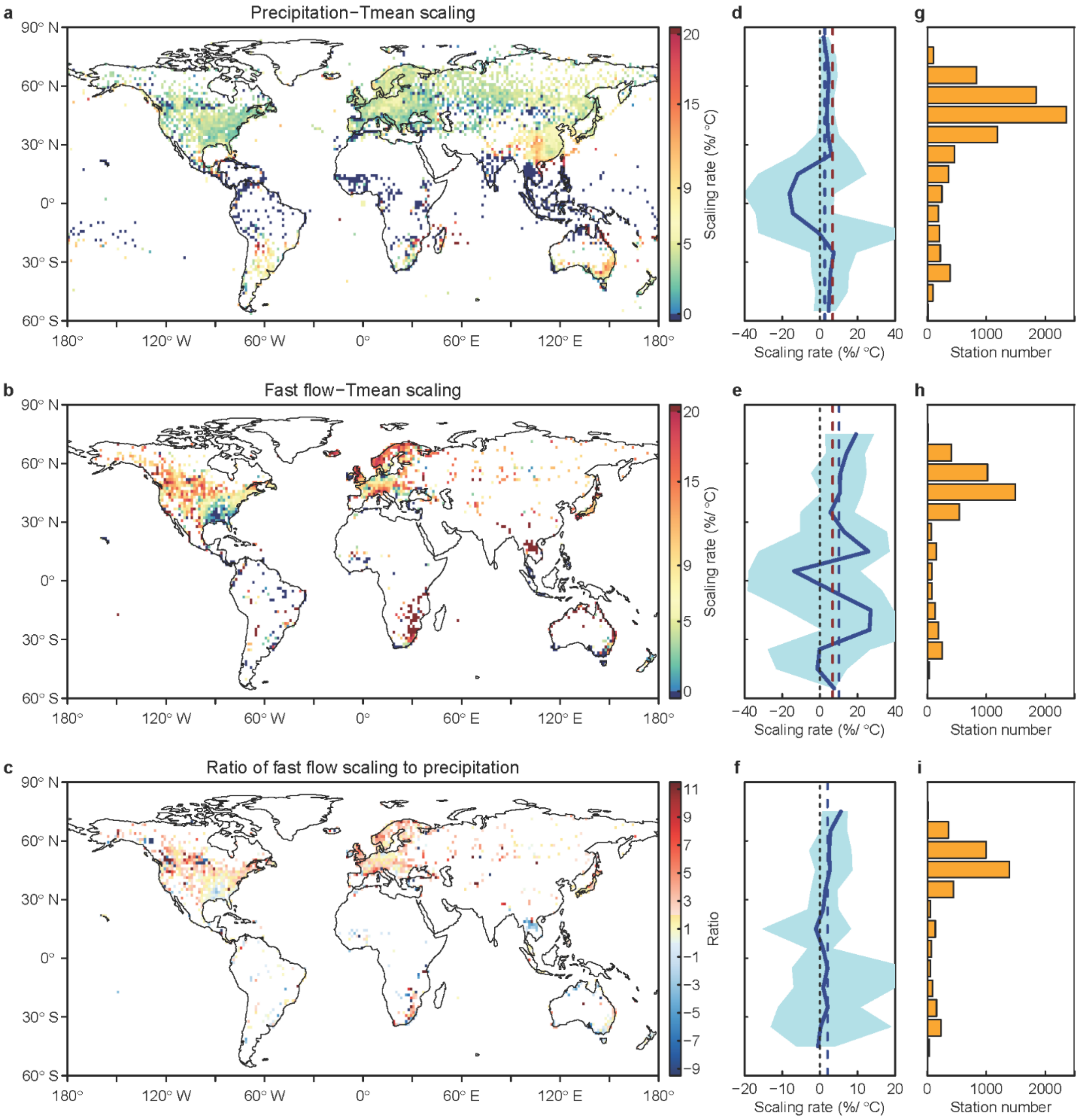

Supplementary Figure 24 | Global scaling results for 95th percentile precipitation and fast flow extremes with same-day Tmean. a, Scaling results of precipitation extremes with same-day Tmean. **b**, Scaling results of fast flow extremes with same-day Tmean. **c,** Ratio of fast flow to precipitation scaling with Tmean. **d-f**, Zonal results based on precipitation scaling, fast flow scaling and ratio, respectively. **g-i**, Used stations for precipitation scaling, fast flow scaling and ratio, respectively. Solid blue lines indicate the median scaling rate or ratio in each latitude band, and the shading shows the associated 90% confidence intervals. Dashed blue lines indicate the global average values, and dashed red lines in **d-e** indicate C-C scaling.

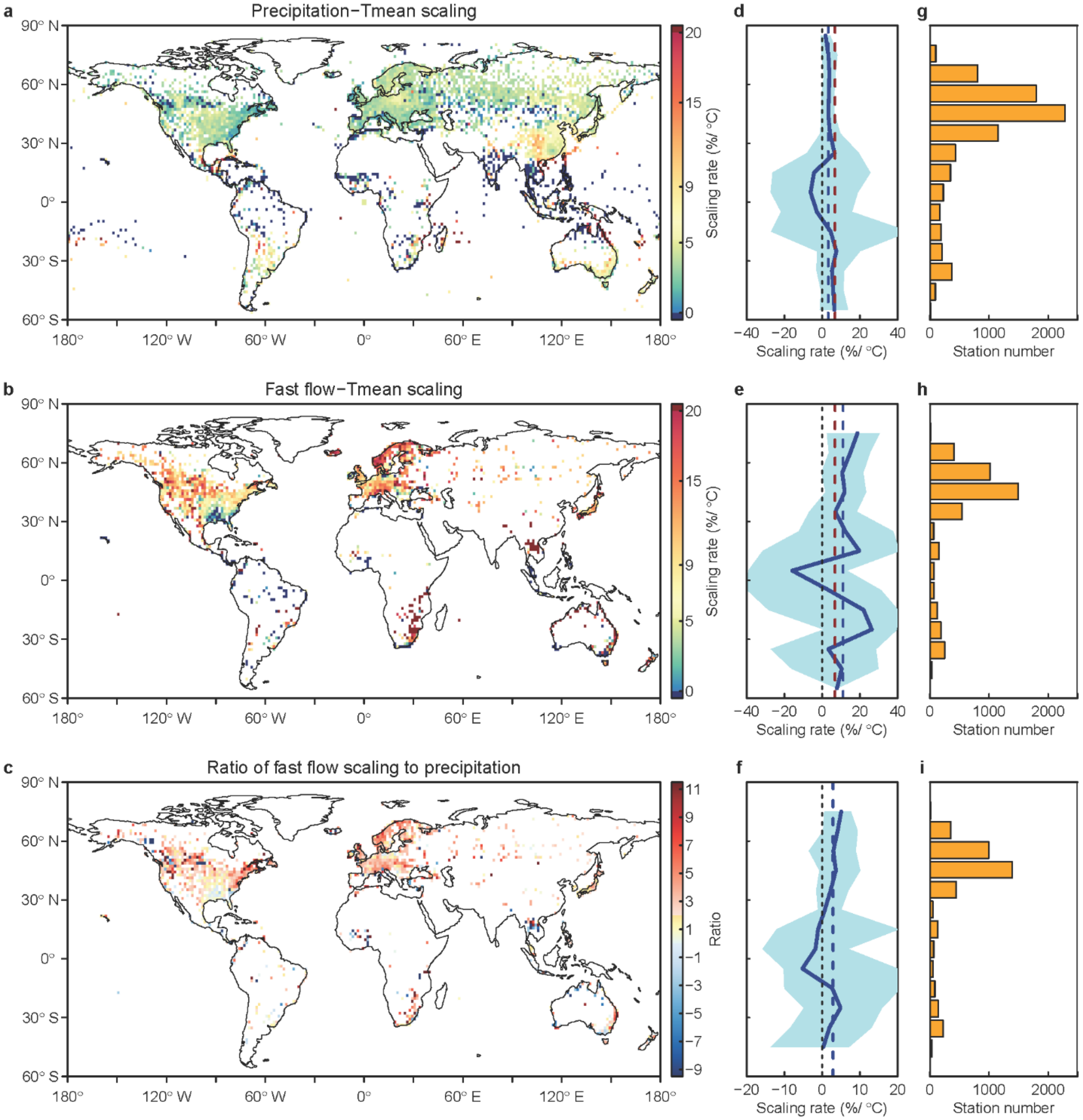

Supplementary Figure 25 | Global scaling results for 95th percentile precipitation and fast flow extremes with previous-day Tmean. a, Scaling results of precipitation extremes with previous-day Tmean. **b**, Scaling results of fast flow extremes with previous-day Tmean. **c,** Ratio of fast flow to precipitation scaling with Tmean. **d-f**, Zonal results based on precipitation scaling, fast flow scaling and ratio, respectively. **g-i**, Used stations for precipitation scaling, fast flow scaling and ratio, respectively. Solid blue lines indicate the median scaling rate or ratio in each latitude band, and the shading shows the associated 90% confidence intervals. Dashed blue lines indicate the global average values, and dashed red lines in **d-e** indicate C-C scaling.

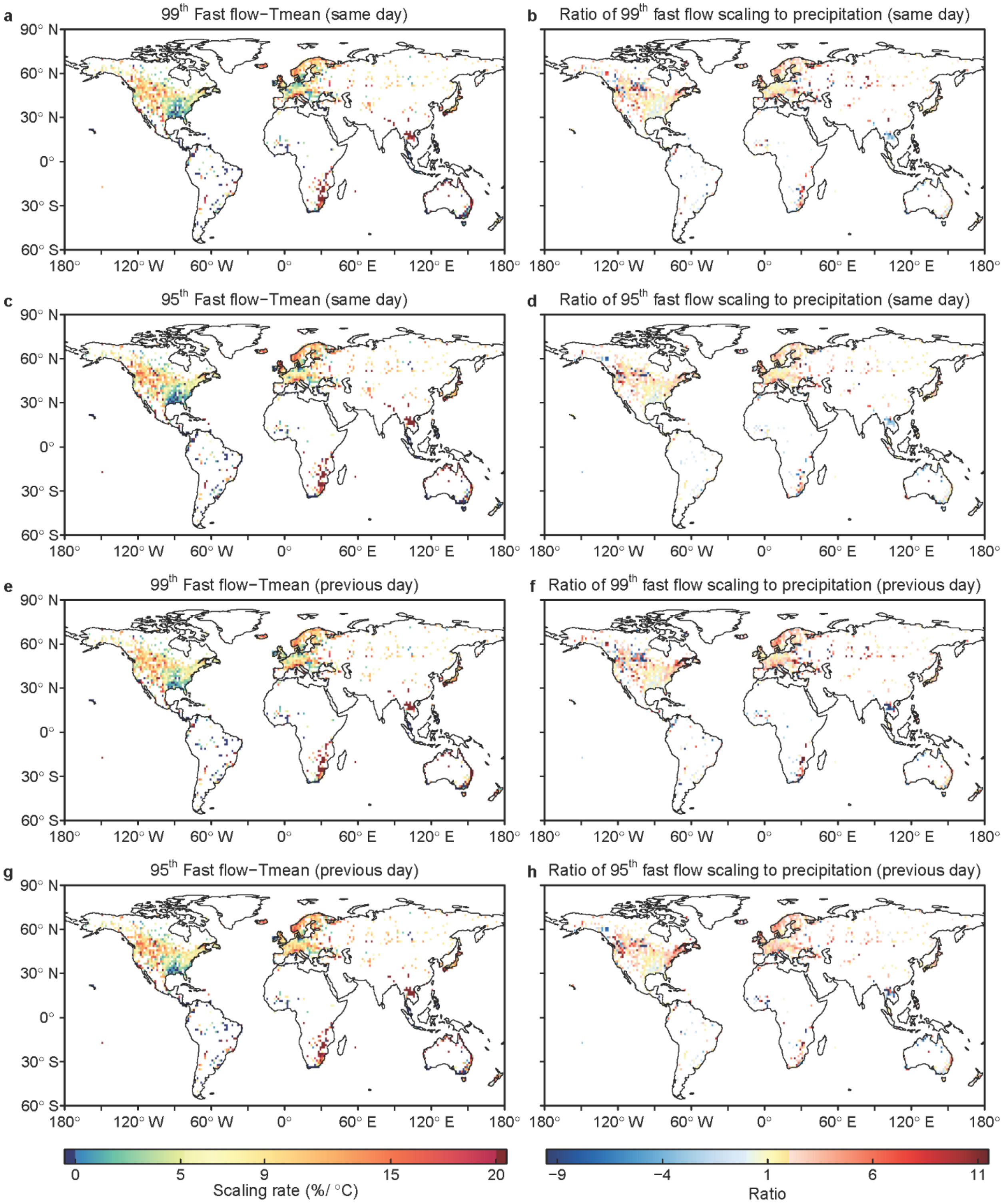

Supplementary Figure 26 | Global scaling results for fast flow extreme with Tmean and the ratio of fast flow to precipitation scaling. The fast flow in this map is derived by a baseline of 25th percentile runoff for non-extreme conditions in each temperature bin. a-d, same-day Tmean with 99th extreme scaling (a) and corresponding ratio to precipitation scaling (b),with 95th extreme scaling (c) and ratio(d). e-f, previous-day Tmean with 99th extreme scaling (e) and corresponding ratio to precipitation scaling (f),with 95th extreme scaling (g) and ratio(h).

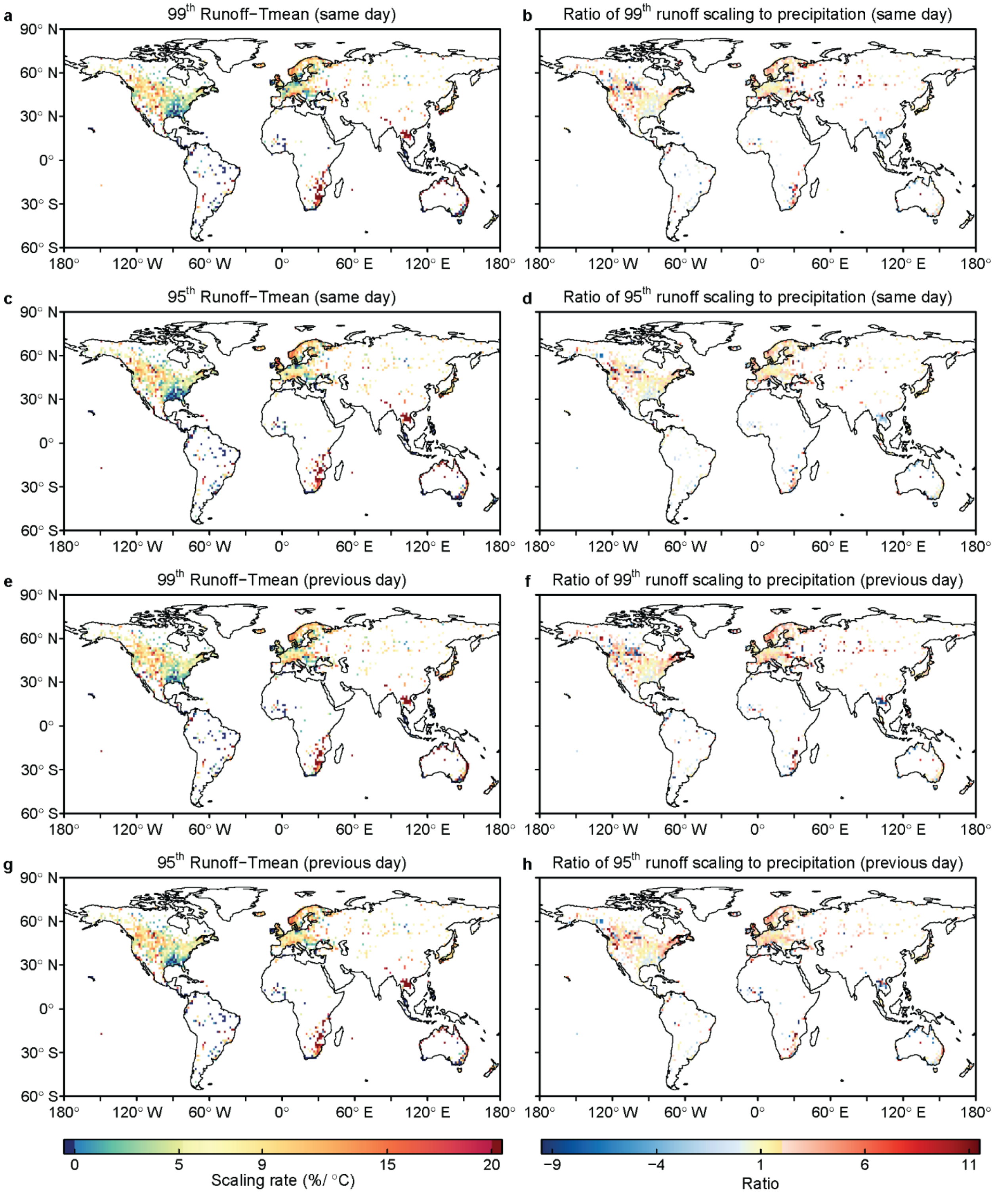

Supplementary Figure 27 | Global scaling results for percentile 99th runoff extreme with Tmean and the ratio of runoff to precipitation scaling. a-d, same-day Tmean with 99th extreme scaling (**a**) and corresponding ratio to precipitation scaling (**b**),with 95th extreme scaling (**c**) and ratio(**d**). **e-f**, previous-day Tmean with 99th extreme scaling (**e**) and corresponding ratio to precipitation scaling (**f**),with 95th extreme scaling (**g**) and ratio(**h**).

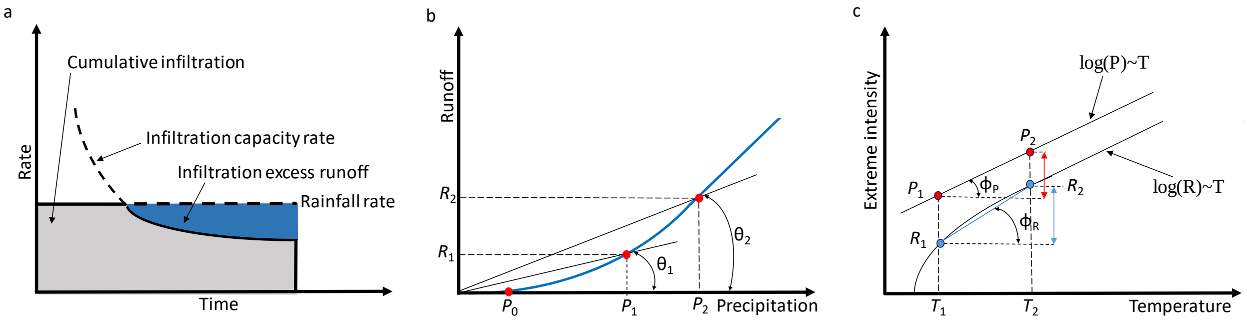

Supplementary Figure 28 | Schematic showing cumulative infiltration and infiltration excess runoff (a), runoff as function of precipitation (b), relationship between runoff/precipitation extremes and temperature (c). P_0 indicates the smallest rain intensity generating infiltration-excess runoff, the runoff coefficients at precipitation events (P_1, P_2) are β_1=tan(θ_1) and β_2=tan(θ_2), respectively. The scaling rates of runoff ($\boldsymbol{\alpha_R}$) and precipitation ($\boldsymbol{\alpha_P}$) are tan(ϕ_R) and tan(ϕ_P), respectively. The red bar in Fig. **c** indicates log(P_2)- log(P_1), the blue bar is log(R_2)- log (R_1). As log(R_2/R_1)- log(P_2/ P_1)=log(β_2/ β_1) ≥0, $\boldsymbol{\phi_R \geq \phi_P}$ and thus $\boldsymbol{\alpha_R \geq \alpha_P}$.

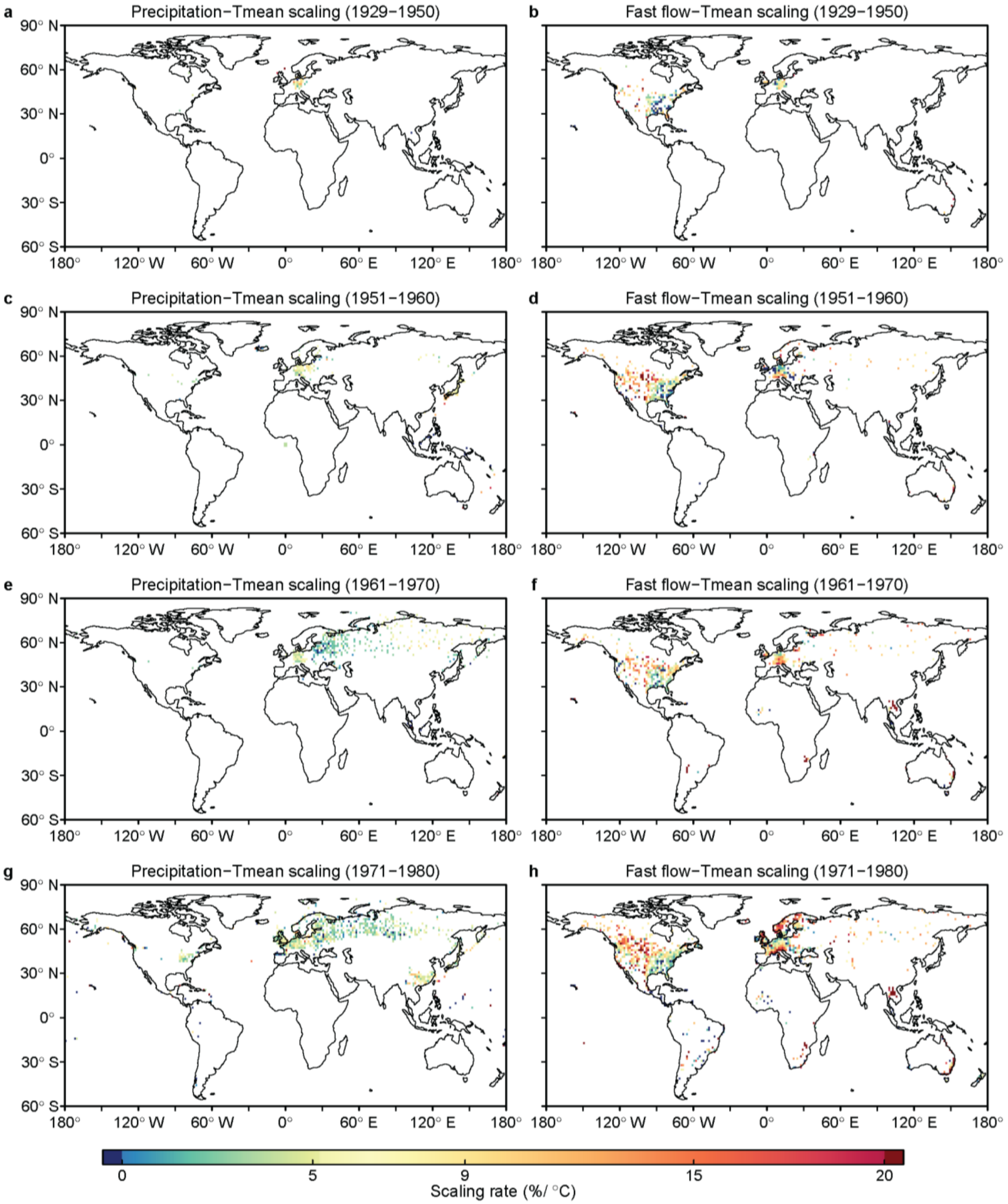

Supplementary Figure 29 | Global scaling rates for 99th percentile precipitation and fast flow extremes with same-day Tmean. The results are estimated under different periods, for 1929-1950 (**a-b**), for 1951-1960 (**c-d**), for 1961-1970 (**e-f**), for 1971-1980 (**g-h**).

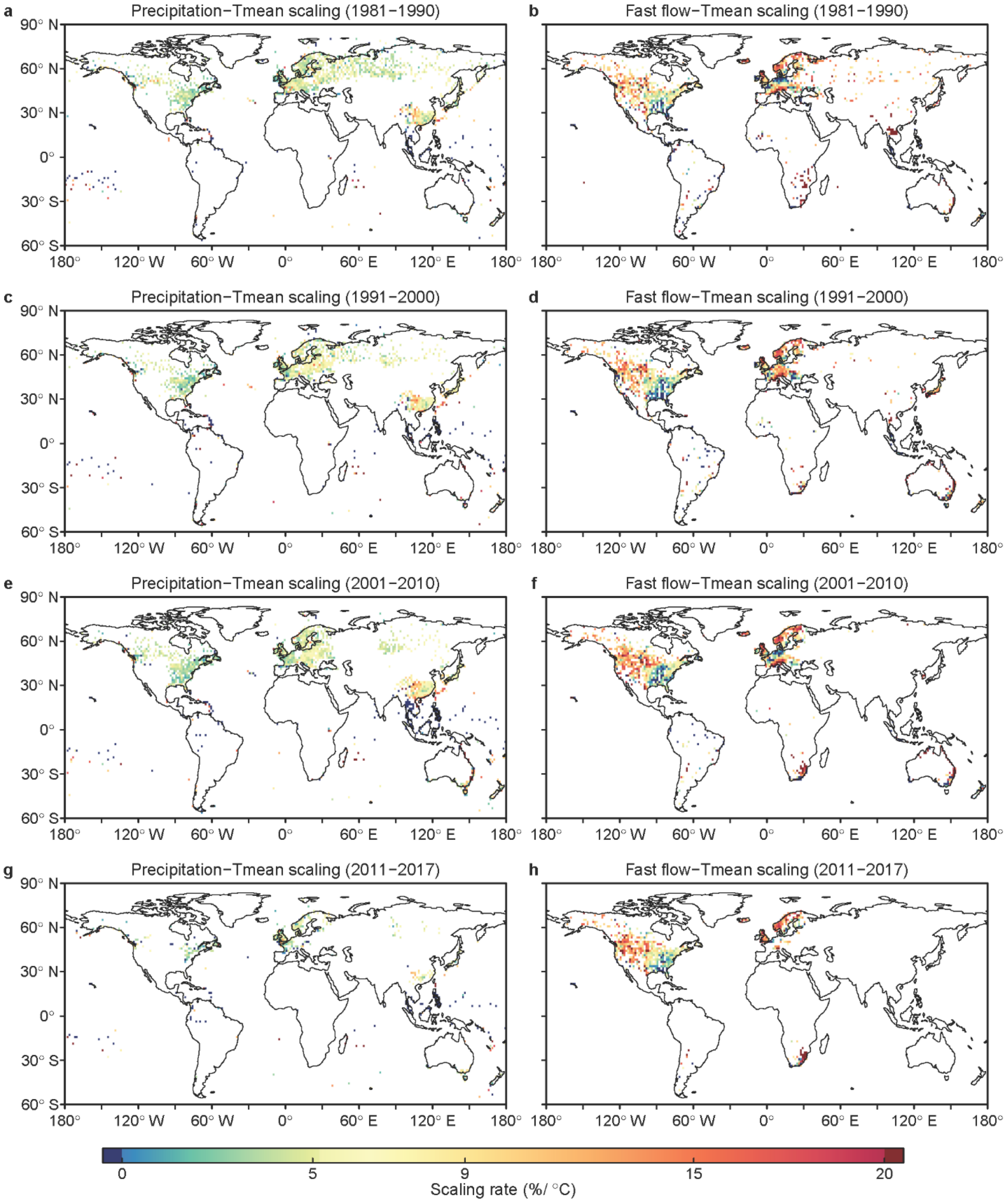

Supplementary Figure 30 | Global scaling rates for 99th percentile precipitation and fast flow extremes with same-day Tmean. The results are estimated under different periods, for 1981-1990 (**a-b**), for 1991-2000 (**c-d**), for 2001-2010 (**e-f**), for 2011-2017 (**g-h**)

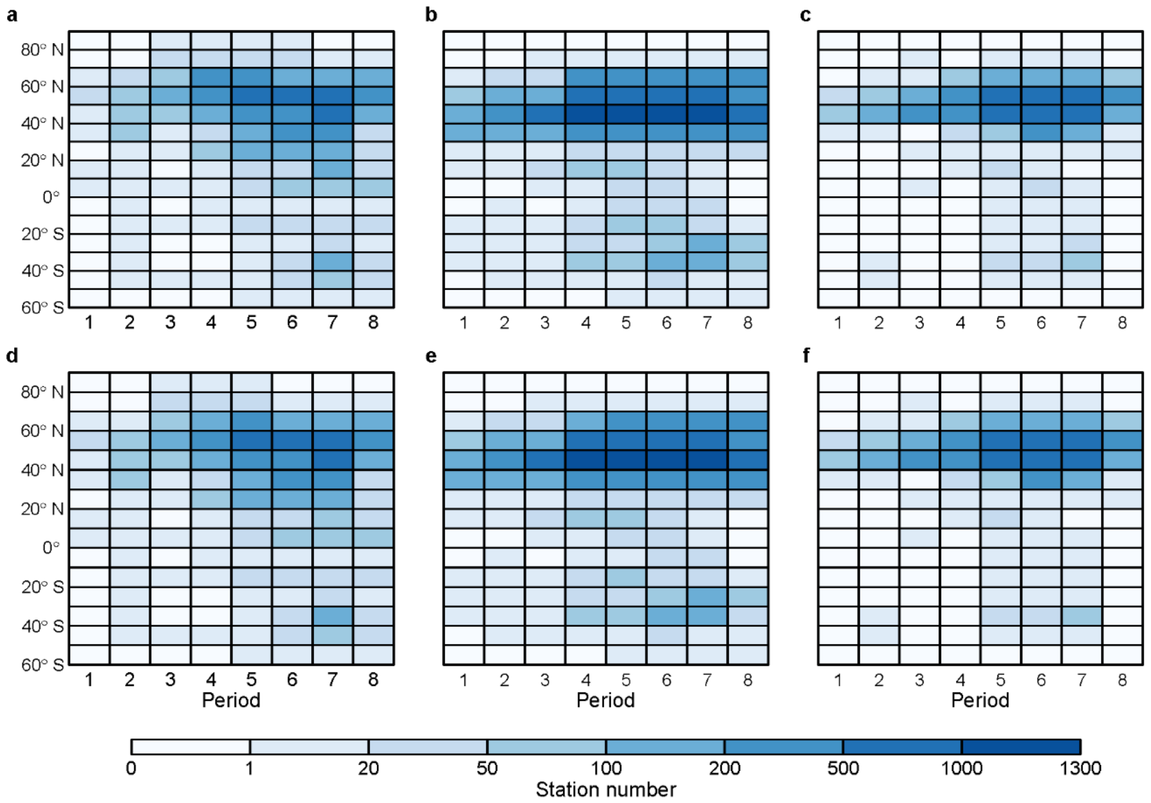

Supplementary Figure 31 | Station number used in each latitude band for different decadal periods. This plot indicates station number used for deriving zonal results in Figure 4. **a**, Rain station numbers for precipitation with same-day Tmean scaling. **b**, Runoff station numbers for fast flow with same-day Tmean scaling. **c**, Runoff station numbers for same-day Tmean scaling ratio. **d**, Rain station numbers for precipitation with previous-day Tmean scaling. **e**, Runoff station numbers for fast flow with previous-day Tmean scaling. **f**, Runoff station numbers for previous-day Tmean scaling ratio. X-axis means 8 periods and Y-axis indicates latitude bands.

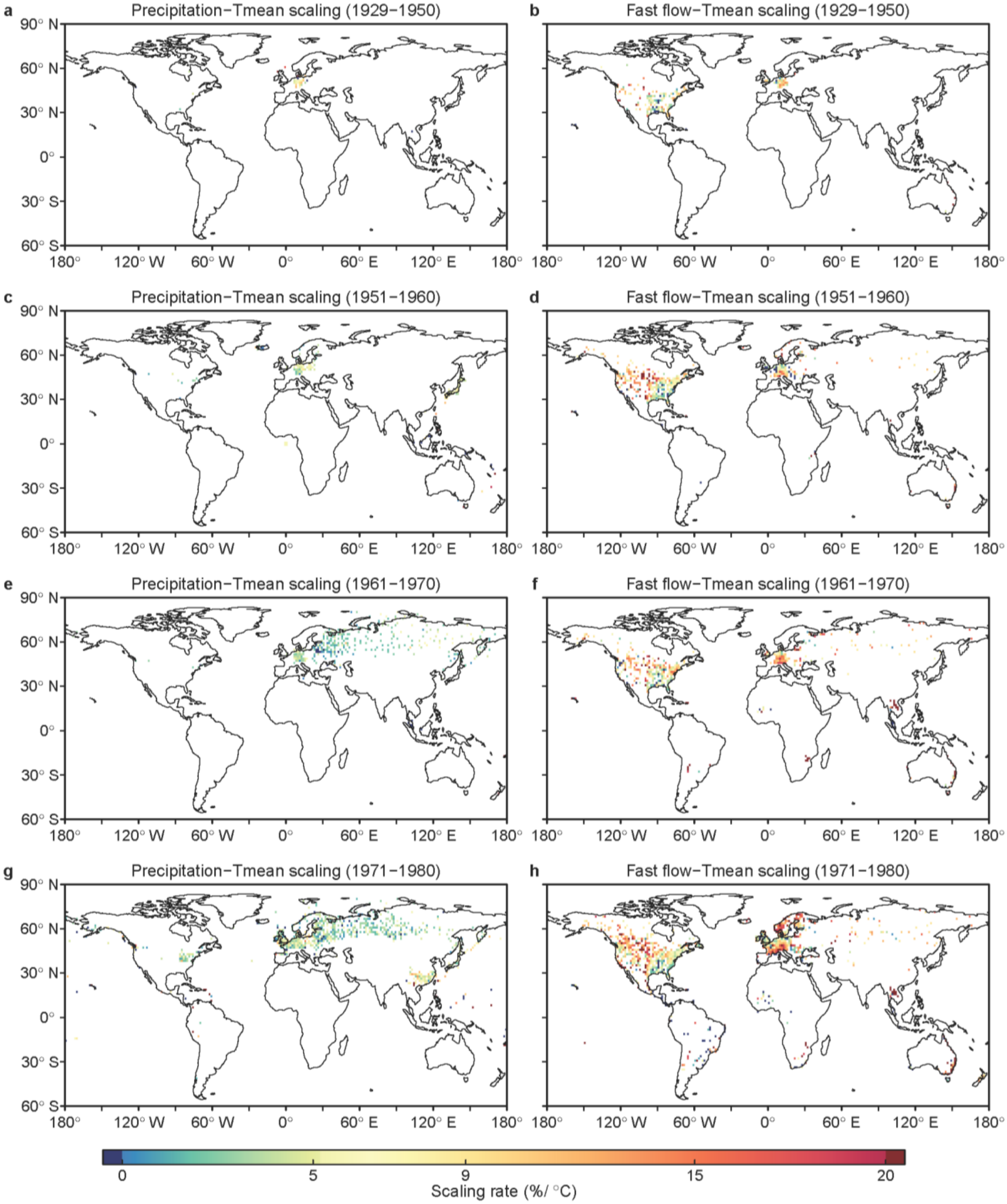

Supplementary Figure 32 | Global scaling rates for 99th percentile precipitation and fast flow extremes with previous-day Tmean. The results are estimated under different periods, for 1929-1950 (**a-b**), for 1951-1960 (**c-d**), for 1961-1970 (**e-f**), for 1971-1980 (**g-h**).

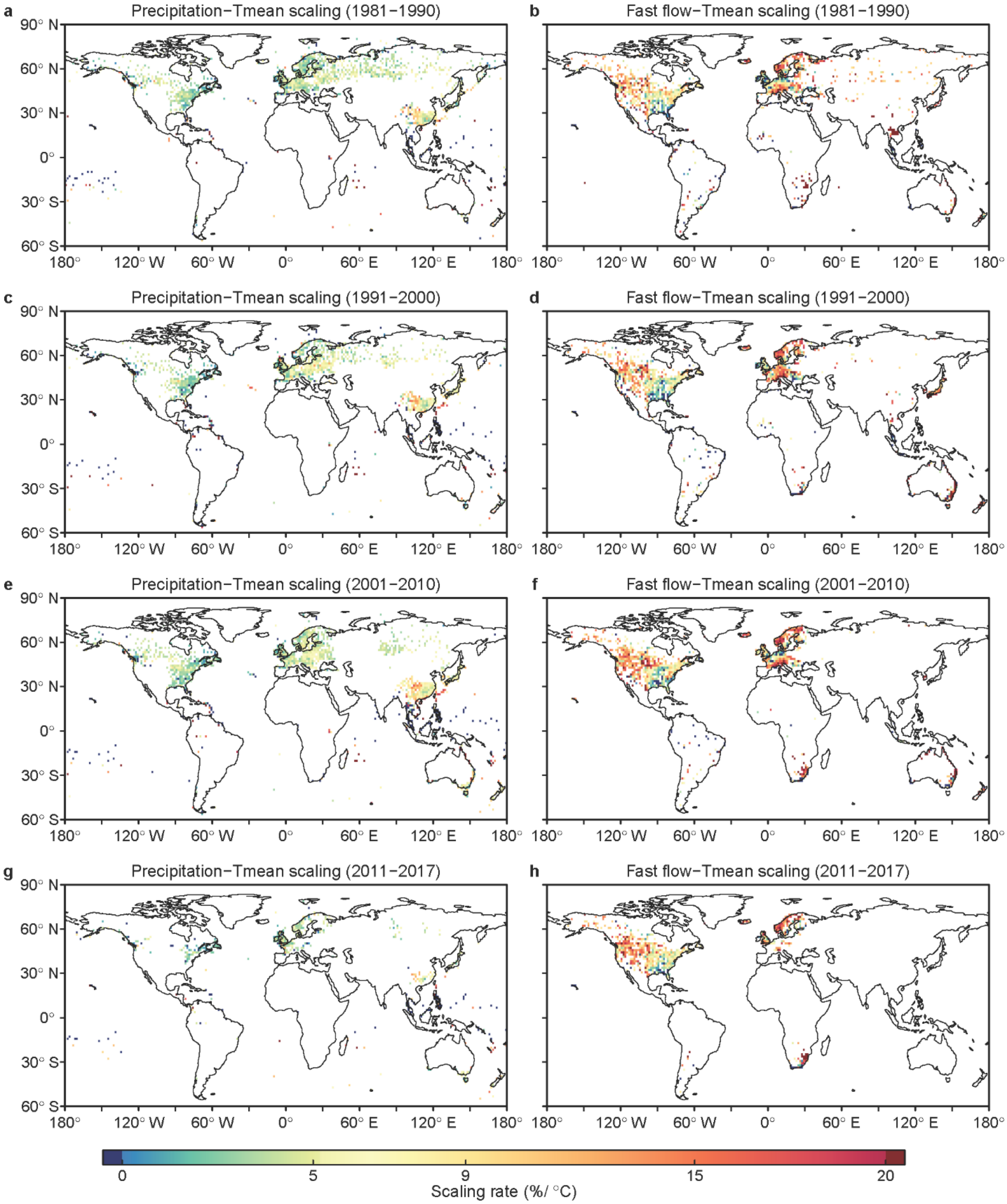

Supplementary Figure 33 | Global scaling rates for 99th percentile precipitation and fast flow extremes with previous-day Tmean. The results are estimated under different periods, for 1981-1990 (**a-b**), for 1991-2000 (**c-d**), for 2001-2010 (**e-f**), for 2011-2017 (**g-h**).

Supplementary Tables

Supplementary Table 1 | Summary of global average scaling rates under different schemes.

Scheme	Temp	Prep 99th	Prep 95th	Runoff 99th	Runoff 95th	[a]Fastflow 99th	[a]Fastflow 95th	[b]Fastflow 99th	[b]Fastflow 95th
Same-day	Tmean	2.96	2.58	8.34	8.25	10.51	10.23	9.14	9.05
	Tmax	3.00	2.69	4.87	4.89	6.21	7.03	5.53	5.46
	Tmin	3.29	3.34	9.14	8.78	11.49	11.16	9.95	9.82
Previous-day	Tmean	3.46	3.21	9.05	8.66	11.18	11.06	9.86	9.76
	Tmax	3.00	2.71	5.45	5.33	6.95	7.74	6.13	6.05
	Tmin	3.91	4.13	9.40	9.05	11.59	11.31	10.15	10.40

Note: [a] indicates fast flow derived by the recursive digital filter method.
[b] indicates fast flow derived by a baseline of 25th percentile runoff for non-extreme conditions in each temperature bin.
